# Effect of nursing interventions on the rehabilitation of patients after knee and hip replacements

**DOI:** 10.1590/1980-220X-REEUSP-2025-0184en

**Published:** 2025-09-15

**Authors:** Qing Sheng, Guangling Wang, Pengfei Wang

**Affiliations:** 1 Jiangsu Province Hospital, The Third Ward of the Department of Orthopedics, Nanjing, China.; 2 Huai’an Economic and Technological Development Zone Hospital, Department of Orthopedics, Huai’an, Jiangsu, China.

**Keywords:** Nursing Care, Rehabilitation, Patient, Knee Prosthesis, Hip Prosthesis, Cuidados de Enfermagem, Reabilitação, Paciente, Prótese do Joelho, Prótese de Quadril

## Abstract

**Objective:**

This systematic review and meta-analysis aimed to assess the effect of nursing interventions, considering their importance in the rehabilitation of patients undergoing knee and hip replacement.

**Methods:**

Three databases were systematically searched. Two researchers carefully reviewed the full texts of the selected studies and extracted the required data. Further, a meta-analysis was conducted.

**Results:**

Of the 19 studies reviewed, 17 revealed that nursing interventions can improve the rehabilitation of patients after knee and hip replacement operations. Among patients with hip replacements, the results indicated that nursing interventions can significantly increase the Harris hip score and activities of daily living compared with usual care. Among patients with knee replacements, the results revealed that nursing interventions can significantly improve range of motion and knee function and reduce knee pain compared with usual care.

**Conclusions:**

Evidence reveals that individualized care and evidence-based strategies combined with various exercises and training provided by a multidisciplinary team significantly influence the physical rehabilitation among patients undergoing knee and hip replacement.

## RESUMO

**Objectivo:**

Esta revisão sistemática e meta-análise teve como objectivo avaliar o efeito das intervenções de enfermagem, considerando a sua importância na reabilitação dos doentes submetidos a artroplastia do joelho e da anca.

**Métodos:**

Foram sistematicamente pesquisadas três bases de dados. Dois investigadores analisaram cuidadosamente os textos completos dos estudos selecionados e extraíram os dados necessários. Além disso, foi conduzida uma meta-análise.

**Resultados:**

Dos 19 estudos revistos, 17 revelaram que as intervenções de enfermagem podem melhorar a reabilitação dos doentes após cirurgias de artroplastia do joelho e da anca. Entre os doentes com artroplastia da anca, os resultados indicaram que as intervenções de enfermagem podem aumentar significativamente o score de Harris para a anca e as atividades da vida diária, em comparação com os cuidados habituais. Entre os doentes com artroplastia do joelho, os resultados revelaram que as intervenções de enfermagem podem melhorar significativamente a amplitude de movimento e a função do joelho, bem como reduzir a dor no joelho, em comparação com os cuidados habituais.

**Conclusões:**

As evidências revelam que os cuidados individualizados e as estratégias baseadas na evidência, combinados com diversos exercícios e treinos fornecidos por uma equipa multidisciplinar, influenciam significativamente a reabilitação física entre os doentes submetidos a artroplastia do joelho e da anca.

## INTRODUCTION

Knee replacement surgery involves replacing the worn-out articular surfaces of the knee joint with artificial implants to relieve pain and restore joint function^(1)^. This surgery technique can help in disease treatment, including knee osteoarthritis, rheumatoid arthritis, and traumatic arthritis^(2)^. Moreover, artificial hip replacement surgery serves as a treatment method for some diseases, such as femoral head avascular necrosis and femoral neck fractures^(3)^. This technique is a popular and effective treatment option for various hip disorders, and it is predominantly used in medical settings^(4)^. Thus, these surgical procedures significantly reduce discomfort and improve the quality of life of patients^(5)^.

However, knee and hip replacements provide considerable advantages, which can be associated with the risks and potential complications such as significant trauma, blood loss, and discomfort^(6,7)^. Consequently, these procedures may cause postoperative pain, limited range of motion, decreased walking speed, diminished muscle strength and physical abilities, impaired stair-climbing abilities, and increased risk of falls. These issues affect the resumption of daily activities and the quality of life of patients^(8)^.

Therefore, the importance of postoperative rehabilitation cannot be ignored, although surgeries may accelerate the return to daily activities^(9)^. Early rehabilitation programs affect long-term functional outcomes and life quality of patients^(10)^. Recent evidence has highlighted the clinical significance of nursing interventions as rehabilitation programs^(11,12)^. Such interventions improve the success of surgical outcomes, optimize the treatment process, reduce postoperative complications, and enhance the quality of life of patients^(13,14,15)^.

Various nursing interventions are available for the rehabilitation of patients undergoing surgical operations. These nursing interventions have been designed based on different approaches. For instance, patient-centered nursing care considers the unique needs of patients when making decisions^(16)^. In contrast, the self-care deficit theory focuses on the self-care capacity of an individual^(17)^. Comprehensive, individualized, and high-quality nursing practices contribute to patient-centered care. Strategies, such as programs led by nurses, telephonic assistance, and educational plans, are associated with the self-care deficit theory. Therefore, different nursing interventions may be related to various effectiveness in the rehabilitation of patients.

Considering the importance of nursing interventions in the rehabilitation of patients undergoing knee and hip replacement, the effectiveness of various nursing interventions needs to be specified. Hence, useful nursing interventions are identified for each of the rehabilitation outcomes. Therefore, a comprehensive study is warranted to summarize the results of previous studies. Zhang et al.^(18)^, in a systematic review and meta-analysis, investigated the effectiveness of improved postoperative recovery in perioperative nursing care among elderly patients with hip and knee arthroplasty^(18)^. Further, Wang et al.^(19)^, in a systematic review and meta-analysis, assessed the effect of preoperative exercise intervention on rehabilitation after total knee arthroplasty^(19)^. However, the procedures and effectiveness of postoperative interventions vary compared with preoperative interventions. Therefore, the effect of postoperative interventions needs to be comprehensively studied. Moon et al., in a systematic review and meta-analysis, investigated the effectiveness of postoperative interventions among patients with total knee/hip replacement, but they only focused on the effects of nurse-led pain management interventions^(20)^. Meanwhile, other types of postoperative nursing need to be considered. Therefore, to address these gaps, the present systematic review and meta-analysis aimed to assess the effect of various nursing interventions on the physical rehabilitation of patients after knee and hip replacement.

## METHOD

This review and meta-analysis study assessed the effectiveness of various physical nursing interventions on the recovery process of individuals undergoing knee and hip replacement surgeries. Therefore, the research question was “whether nursing interventions can affect the rehabilitation of patients after knee and hip replacements.” The present review followed the Joanna Briggs Institute (JBI) protocol. The guideline of the Preferred Reporting Items for Systematic Reviews and Meta-Analyses (PRISMA) was utilized to write the article^(21)^. Further, this comprehensive review and meta-analysis is formally documented in the PROSPERO registry (CRD420251039484).

### Eligibility Criteria

In this research, the authors did not consider restrictions associated with ethnicity, nationality, gender, age, or comparable factors. The inclusion criteria comprised a wide range of articles published in English. Further, as exclusion criteria, conference papers, notes, conference reviews, book chapters, editorials, brief surveys, letters, and review articles were excluded. Moreover, studies of insufficient quality were eliminated.

### Search Strategy

To achieve the objectives of this study, an extensive and methodical search was conducted across Web of Science, Scopus, and PubMed to find relevant articles that were published up to March 24, 2025. Initial keywords were identified to conduct a systematic search. Articles were then classified based on their semantic and conceptual similarities (Table 1). Table S1 represents the search strategy employed in the present study

**Table 1:** Categorization of keywords based on semantic and conceptual similarities – Huai’an, JS, China, 2024.

Categories	Related keywords
Nursing Care and Interventions	Nursing care, nursing process, perioperative nursing, postoperative care, nurse-led, care intervention, nursing management, continuous nursing, care intervention, nursing support, nonpharmacologic intervention
Surgical Procedures and Implants	“Arthroplasty, replacement, knee,” “arthroplasty, replacement, hip,” “knee prosthesis,” “hip prosthesis,” “knee replacement,” “hip replacement,” “total knee arthroplasty,” “total hip arthroplasty,” “joint replacement,” “knee surgery,” “hip surgery,” “prosthetic knee,” “prosthetic hip”
Rehabilitation and Recovery	Rehabilitation,” “rehabilitation nursing,” “recovery of function,” “physical therapy modalities,” “functional recovery,” “physical rehabilitation,” “mobility,” “range of motion,” “functional outcome,” “postoperative recovery,” “physical function,” “quality of life,” “exercise therapy”

These keywords were systematically combined using Boolean operators to establish a tailored search strategy. This strategy was then implemented across databases to collect pertinent literature.

### Study Selection

All retrieved articles were imported into EndNote. Duplicates were subsequently identified and removed. Two researchers (Q.S. and P.W.) independently screened the remaining articles by assessing their titles and abstracts. Irrelevant studies based on the inclusion and exclusion criteria were then removed. Further, both researchers (Q.S. and P.W.) reviewed the references of the selected articles to uncover any pertinent studies. Afterward, the full texts of the articles were assessed to determine relevant papers.

### Quality Evaluation

The quality of the selected studies was evaluated using the JBI critical appraisal tools designed for randomized controlled trials (RCTs), cohort, case-control, and quasi-experimental studies. The JBI checklist is well-regarded for assessing the methodological rigor of studies^(22)^. JBI criteria for RCT assessment included the use of true randomization utilized for participant group assignment, treatment group allocation concealment, treatment group similarity at baseline, participants blinded to treatment assignment, information of persons providing treatment blinded to treatment assignment, treatment similarity in groups managed other than the intervention of interest, outcome assessors blinded to treatment assignment, outcome measurement similarity for treatment groups, outcomes measurement with a reliable method, follow up completion, participant analysis in the groups to which they were randomized, statistical analysis appropriateness, and trial design appropriateness and consideration of any deviations from the standard RCT design in the analysis. JBI criteria was employed to assess the cohort studies using the criteria: similarity between two groups, similarity of exposure measurement, validity and reliability of exposure measurement, identification of confounding factors, strategies to deal with confounding factors, information of participants about the outcome at the start of the study, validity and reliability of outcomes measurement, sufficiency of follow-up time, completion of follow up, strategies to address incomplete follow up, and appropriateness of statistical analysis. Case-control studies were assessed using the criteria: comparability of case and control groups, appropriate matching of case and control groups, similarity of criteria for identifying case and control groups, exposure measurement with a standard, valid and reliable method, exposure assessment with a similar method for case and control groups, identifying confounding factors, strategies to deal with confounding factors, adequacy of exposure period, and appropriateness of statistical analysis. Semi-experimental studies were assessed using the criteria: cause–and–effect clearness, control group existence, similar comparisons among participants, similar exposure among participants, outcome multiple measurements, similar comparisons of outcomes of participants, reliable measurement of outcomes, follow-up status, and appropriateness of statistical analysis. Each checklist was completed, and positive responses were summed up to calculate a total score. Based on these scores, the studies were then categorized into three quality groups: low, moderate, and high.

### Data Extraction

Two authors (Q.S. and P.W.) independently performed the task using a standardized template to ensure the thoroughness and precision of the data extraction process. This template was developed to collect a wide range of details, including the author(s) and publication year, country, study design, sample size, gender, age, nursing intervention type, nursing intervention duration, service providers, outcome assessment method, body region, results, and outcomes. This step ensured that all gathered information was accurate, complete, and aligned with the predefined criteria.

### Data Synthesis and Analysis

In this investigation, the agreement between the findings of two researchers (Q.S. and P.W.) was assessed using Cohen’s kappa test^(23)^. This value in the present study was obtained by 0.91, which was acceptable. A meta-analysis, in addition to the descriptive results, was conducted on the values of the standardized mean differences (SMDs) associated with various rehabilitation outcomes reported in some studies. The third version of the comprehensive meta-analysis software was used for data analysis. Moreover, GRADE pro software was utilized to assess outcome certainty.

## RESULTS

### Search Results and Study Selection

**Figure 1:**
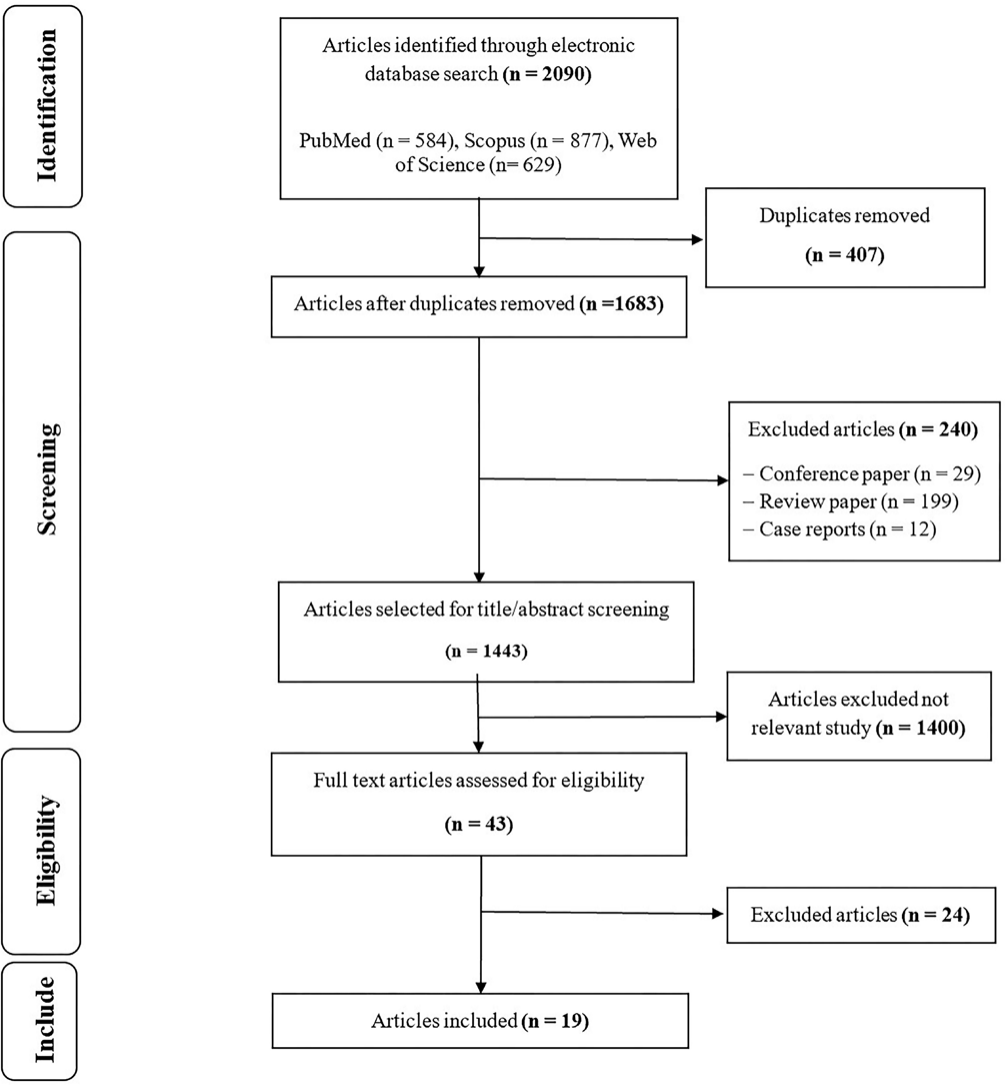
PRISMA flow diagram.

The databases yielded a total of 2,090 articles. Of these, 407 duplicate articles were removed in EndNote software, resulting in 1,683 articles available for screening. Applying the exclusion criteria, 240 studies were removed, comprising 29 conference papers, 12 case reports, and 199 review articles, leaving 1,443 articles for further assessment. Of these, 1,400 irrelevant studies were excluded after the title and abstract screening process. Ultimately, 43 papers remained for full-text review, of which 19 were included in the study. These papers were conducted between 2004 and 2025, mostly after 2020 (14 [73.6%] studies). Figure 1 illustrates the flow diagram of PRISMA.

### Specification of the Articles

**Figure 2:**
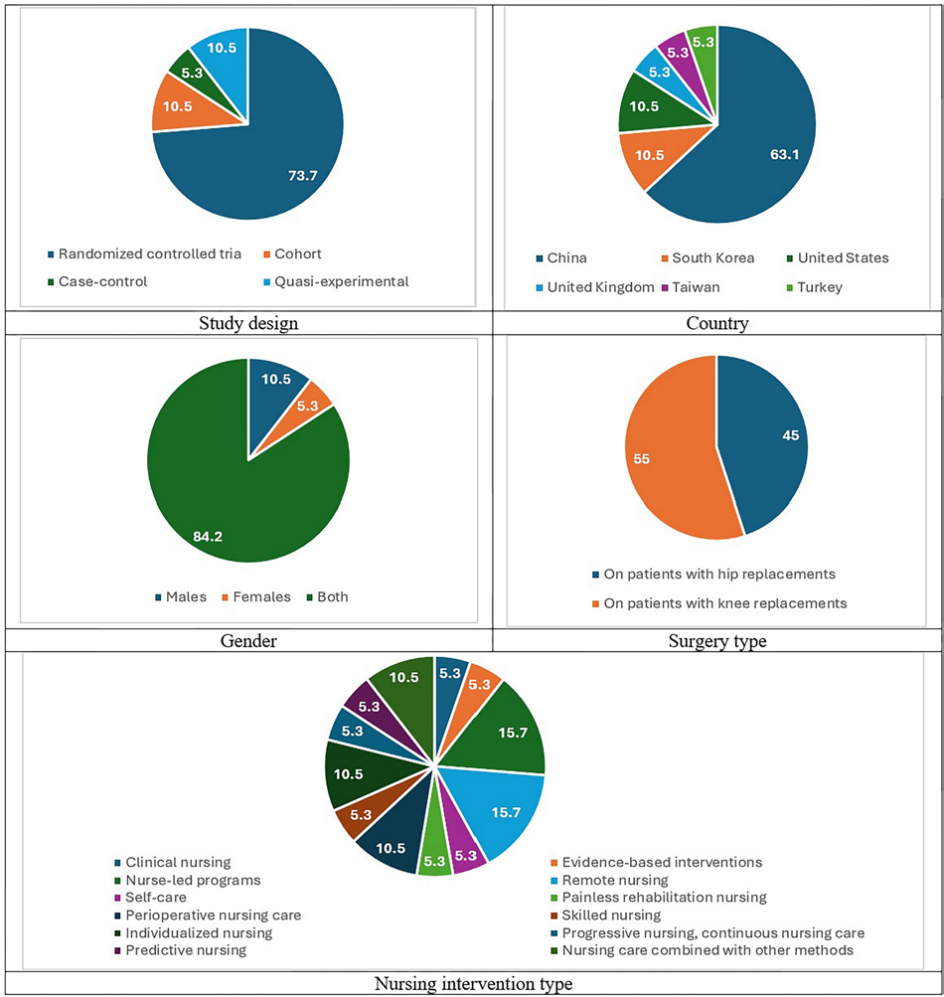
Statistical distribution of the reviewed studies based on various characteristics.

Table 2 shows the total data from the reviewed articles. Figure 2 illustrates the statistical distribution of the reviewed studies according to various characteristics. The design of reviewed studies was RCT (14 studies), cohort (2 studies), case-control (1 study), and quasi-experimental (2 studies). These studies were conducted in China (12 studies), South Korea (2 studies), the United States (2 studies), the United Kingdom (1 study), Taiwan (1 study), and Turkey (1 study). The studies were performed on males (2 studies), females (1 study), and both genders (16 studies). The mean age of participants ranges from 38.40 to 80.60 years. These studies included 2,234 participants.

**Table 2:** Details of the reviewed studies – Huai’an, JS, China, 2024.

Author (year)	Country	Study type	Sample size	Gender	Mean age (year)	Nursing intervention type	Nursing intervention duration (months)	Service providers	Outcome assessment method	Region	Results	Outcome	Quality
Zhang and Xiao (2020)^(24)^	China	Randomized controlled trial (RCT)	70	Both	59–60 years	Clinical nursing pathway	2-3 weeks	Multidisciplinary team (nurses, physiotherapists, and surgeons)	Harris hip score (HHS) and satisfaction score	Hip	The intervention improved hip function, shortened recovery time, and increased satisfaction.	HHS: Mean difference (MD): 7.05 (95% confidence interval [CI]: 3.70–10.40) Satisfaction: MD: 2.97 (95% CI: 1.56–4.38)	Q2
Liu et al. (2023)^(25)^	China	Retrospective Cohort Study	80	Males	64.7 years	Enhanced recovery after surgery (ERAS) with multidisciplinary	–	Multidisciplinary team (surgeons, anesthesiologists, nurses, physiotherapists, nutritionists, and pharmacists)	HHS, range of motion (ROM), and complications	Hip and knee	The intervention exhibited a positive effect on rehabilitation outcomes, including functional recovery.	Postoperative complications: No significant difference (risk ratio [RR] ≈ 1, *P* > 0.05) ROM: MD: 20 (95% CI: 10.5–29.5) HHS: MD: 5 (95% CI: 2.62–7.38)	Q2
Yu et al. (2024)^(3)^	China	Prospective comparative clinical trial	80	Both	73.0 years	Elastic stretch traction belt and pain assessment nursing care	6 months	Nurses and medical staff	HHS and SER scale (self-efficacy)	Hip	Improved rehabilitation outcomes in hip function, self-efficacy, and quality of life.	HHS: MD: 16.09 (95% CI: 8.45–23.73) Self-efficacy: MD: 8.22 (95% CI: 4.32–12.12)	Q1
Zhao et al. (2022)^(26)^	China	RCT	124	Both	74.29	Painless rehabilitation nursing	6 months	Nurses	Knee circumference, knee pain, knee function, agitation, and sleep duration	Knee	The observation group demonstrated reduced pain, improved knee function, and lower agitation rates compared with the control group.	Knee pain: MD: −1.25 (95% CI: 0.66–1.84) Knee motion: MD: 12.06 (95% CI: 6.33–17.79)	Q3
Sun et al. (2021)^(27)^	China	RCT	80	Both	38.48	Perioperative nursing care	1 month	Orthopedic nurses	Harris knee joint function scale and visual analog scale (VAS)	Knee	Significant improvement in pain scores and nursing satisfaction in the study group compared with the control group.	Knee function score: MD: 10.3 (95% CI: 5.41–15.19) Pain score: MD: −1.8 (95% CI: 0.95–2.66)	Q1
Chang et al. (2024)^(8)^	Taiwan	RCT	52	Both	73.44	Nurse-led hybrid teaching program	4 months	Nurses and physiotherapists	Quadriceps strength and knee injury and osteoarthritis outcome score (KOOS)	Knee	A hybrid teaching program improved rehabilitation outcomes, including muscle strength and knee function.	Quadriceps strength: MD: 5.2 (95% CI: 2.73–7.67) KOOS score: MD: 12.4 (95% CI: 6.51–18.29)	Q2
Li et al. (2021)^(28)^	China	RCT	86	Both	–	ERAS nursing combined with limb training	3 months	Nurses and orthopedic physicians	VAS, Judet scoring, and the National Institute of Health Stroke Scale	Knee	ERAS nursing combined with limb training enhances rehabilitation outcomes.	Pain score: MD: −1.5 (95% CI: 0.79–2.21) Knee joint function: MD: 8.2 (95% CI: 4.30–12.10) Neurological function: MD: 2.3 (95% CI: 1.21–3.39)	Q1
Ko et al. (2019)^(29)^	South Korea	Quasi-experimental study	37	Both	–	Individualized transitional care program (ITCP)	–	Nurses	Activities of daily living and timed up-and-go scores	Hip	ITCP is effective in reducing functional decline in older adults after hip arthroplasty.	Functional decline (FD): MD: 2.2 (95% CI: 1.15–3.25) Activities of daily living (ADL): MD: 4.4 (95% CI: 2.31–6.49)	Q1
Deane et al. (2018)^(39)^	United Kingdom	RCT	144	Male	69.8	Patient-directed self-management of pain	–	Nurses and the research team	VAS for pain	Knee	Self-medication did not lead to better pain control compared with nurse-managed patients.	Pain: MD: −0.99 (95% CI: 0.11–1.87)	Q1
Kauh et al. (2005)^(30)^	USA	Retrospective Observational Pilot Study	150	Female	80.6	Skilled nursing for geriatric rehabilitation	1 year	Geriatric nurse practitioners and an interdisciplinary team	Rehabilitation outcome measure (ROM) for activities of daily living and mobility.	Hip	This strategy proved to be an efficient approach in improving patient outcomes and minimizing unnecessary use of health care services after a severe illness.	Activities of daily living: MD: 0.41 (95% CI: 0.22–0.60) Mobility: MD: 0.29 (95% CI: 0.15–0.43)	Q1
Losina et al. (2016)^(10)^	USA	RCT	308	Both	66.0	Motivational interviewing (MI)-based postoperative care navigation	6 months (10 calls)	Surgeon, home care nurse, and physical therapist	WOMAC pain score, satisfaction, and ROM.	Knee	Participants in the navigation intervention group did not show more significant functional improvement than those in the control group.	Pain: MD: –3 (95% CI: −7.00–1.00) Satisfaction: MD: 8.00 (95% CI: 4.2–11.8) ROM: MD: 2.00 (95% CI: 1.05–2.95)	Q1
Biricik et al. (2024)^(31)^	Turkiye	RCT	44	Both	66.3	Video-assisted patient education (VPE) on postoperative care, ADLs, and exercises.	3 months	Nurses (primary educators) and physiotherapists	WOMAC score and SF-36	Knee	This intervention improved knee function, enhanced the quality of life, and reduced complications post-TKR.	Pain: MD: −3.5 (95% CI: −5.7 to −1.3) Physical Function: MD: −10.6 (95% CI: −18.7 to −2.5) Physical Role: MD: 30.7 (95% CI: 7.4–54.0)	Q3
Wang et al. (2018)^(32)^	China	RCT	389	Both	55.67	Internet-based home orthopedic care platform (WeChat) with nurse specialist support (rehabilitation monitoring, and appointment scheduling)	6 months	Orthopedic nurses	HHS, Barthel index (BI), and SF-36 scale	Hip	This intervention significantly affected joint function, activities of daily living, and quality of life in the intervention group.	MD: HHS: MD: 6.07 (95% CI: 3.19–8.95) Activities of daily living (BI): 5.02 (95% CI: 2.64–7.40) Quality of life (SF-36): 6.78 (95% CI: 3.56–10.0)	Q1
Pua et al. (2020)^(33)^	China	RCT	76	Both	67.8	Individualized nursing (Interactive nursing, pain management, rehabilitation exercises, psychological support, and complication prevention)	30 months	Clinical nurses	Hospital for Special Surgery (HSS) Score	Knee	Individualized nursing interventions enhanced the recovery of limb functionality and improved the quality of life among patients.	Function score: MD: 1.8 (95% CI: 0.43–3.17)	Q3
Li et al. (2023)^(34)^	China	Prospective non-RCT	115 patients	Both	≥60	Progressive nursing	7 days	Clinical nurses	Knee function and complications	Knee	Progressive nursing improved functional recovery, reduced complications, and enhanced the quality of life.	Knee function score: MD: 1.8 (95% CI: 0.95–2.66)	Q1
Xiao et al. (2024)^(35)^	China	Retrospective case-control	90	Both	45.04	Nurse-led pain management	–	Multidisciplinary team (nurses, anesthesiologists, doctors, rehabilitators, and pharmacists)	ROM, HHS, complications, and satisfaction (NSNS)	Hip	This intervention improved pain management, satisfaction, and joint function and reduced complications.	Pain intervention efficacy: MD: 1.8 (95% CI: 0.94–2.66) ROM: MD: 13.22 (95% CI: 6.94–19.5) Complications: MD: –14.55 (95% CI: – 7.64 to −21.46) Satisfaction: MD: 17.07 (95% CI: 8.96–25.18)	Q2
Bak and Uhm (2024)^(36)^	South Korea	Quasi-experimental	45	Both	66.78	Nurse-led app-based home exercise program (videos and push notifications)	3 months	Nurses, app developers, and physical therapists	VAS (pain), ROM, exercise self-efficacy scale, and NSNS (satisfaction)	Knee	Nursing care improved pain control, ROM, self-efficacy, and satisfaction.	Pain: MD: −2.93 (95% CI: −1.54 to −4.32) ROM: MD: 14.9 (95% CI: 7.82–21.98) Self-Efficacy: MD: 2.81 (95% CI: 1.48–4.14)	Q2
Guo et al. (2022)^(37)^	China	RCT	134	Both	68.40	Continuous nursing care (CNC)	3 months	Nurses, physicians, and rehabilitation therapists	HHS and Barthel index (ADL)	Hip	Continuous nursing care improved hip function and activities of daily living.	HHS: MD: 8.41 (95% CI: 4.42–12.40) Barthel index (ADL): MD: 5.90 (95% CI: 3.10–8.70)	Q1
Guo and Zhang (2025)^(38)^	China	RCT	130	Both	70.5	Predictive nursing (risk assessment-driven)	3 months	Nurses and orthopedic surgical teams	HHS, Barthel index (ADL), complications, and satisfaction scores	Hip	Predictive nursing care was associated with shorter hospital stays, heightened satisfaction levels, enhanced hip function, improved ADL scores, and reduced complications.	HHS: MD: 12.1 (95% CI: 6.35–17.85) Barthel index (ADL): MD: 13.2 (95% CI: 6.93–19.47)	Q1

Nursing interventions included clinical nursing (1 study), evidence-based interventions (1 study), nurse-led programs (3 studies), remote nursing (3 studies), self-care (1 study), painless rehabilitation nursing (1 study), perioperative nursing care (2 study), skilled nursing (1 study), individualized nursing (2 studies), progressive nursing, continuous nursing care (CNC) (1 study), predictive nursing (1 study), and nursing care combined with other methods (2 studies). Further, 9 and 11 studies were conducted on patients with hip and knee replacements, respectively. The time duration of the interventions varied from 7 days to 30 months. In these studies, service providers included general nurses, orthopedic nurses, geriatric nurse practitioners, home care nurses, clinical nurses, physiotherapists, physical therapists, rehabilitation therapists, surgeons, orthopedic physicians, anesthesiologists, nutritionists, pharmacists, rehabilitators, and other medical staff. The most predominant outcomes of rehabilitation were Harris hip score, range of motion, self-efficacy, pain score, knee or hip function, neurological function, activities of daily living, mobility, quality of life, satisfaction score, and complications.

**Figure 3:**
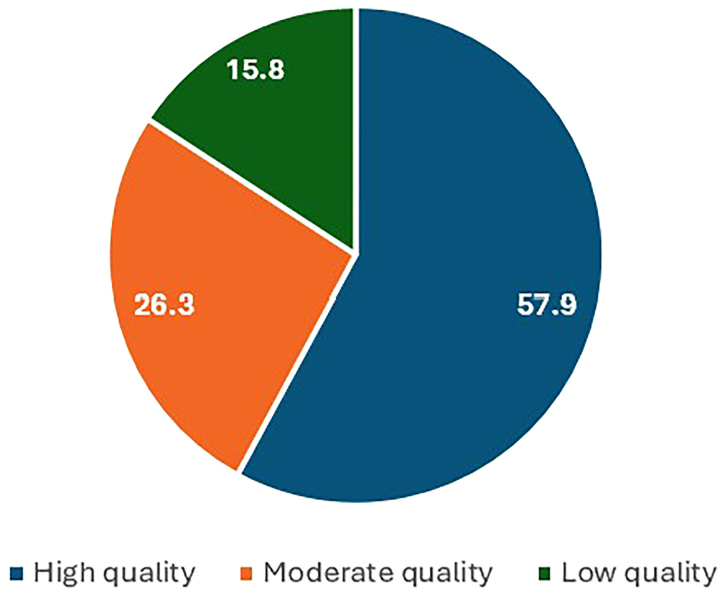
Statistical distribution of the reviewed studies based on quality level.

Figure 3 illustrates the statistical distribution of the reviewed studies according to quality level. Based on the results related to quality assessment of papers, 11, 5, and 3 studies were classified into the group of high, moderate, and low-quality articles, respectively. The results revealed that the quality levels of the reviewed studies could not affect the findings of the present study because only three (15.8%) studies were of low quality.

### Main Findings

Of the 19 reviewed studies, 17 demonstrated that nursing interventions can improve the rehabilitation of patients after knee and hip replacement^(3,8,24–38)^. Two studies exhibited not only significant associations between nursing interventions and rehabilitation in patients with knee replacement^(10,39)^. The type of nursing intervention in these two studies includes remote nursing, comprising patient-directed self-management of pain^(39)^ and motivational interviewing (MI)-based postoperative care navigation^(10)^.

Various rehabilitation outcomes were assessed in the studies. The predominant outcomes assessed in patients with hip replacement included the Harris hip score and activities of daily living, whereas range of motion and knee function were evaluated in patients with knee replacement.

Among the nursing interventions for patients with hip replacements, elastic stretch traction belt and pain assessment nursing care performed by nurses and medical staff during 6 months (MD: 16.09; 95% confidence interval [CI]: 8.45– 23.73)^(3)^, predictive nursing conducted by nurses and orthopedic surgical teams during 3 months (MD: 12.1; 95% CI: 6.35– 17.85)^(38)^, and CNC provided by nurses, physicians, and rehabilitation therapists during 3 months (MD: 8.41; 95% CI: 4.42–12.40)^(37)^ had highest MDs in harries hip score. Further, predictive nursing showed the greatest MDs in activities of daily living (MD: 13.2; 95% CI: 6.93–19.47)^(38)^.

Among the nursing interventions for patients with knee replacements, perioperative nursing care conducted by orthopedic nurses in 1 month and enhanced recovery after surgery (ERAS) nursing combined with limb training performed by nurses (MD: 10.3; 95% CI: 5.41–15.19)^(27)^ and orthopedic physicians in 3 months (MD: 8.2; 95% CI: 4.30–12.10)^(28)^ exhibited highest MDs in knee motion. Moreover, ERAS with multidisciplinary performed by a multidisciplinary team (nurses, surgeons, anesthesiologists, nutritionists, physiotherapists, and pharmacists) in 12 months (MD: 20; 95% CI: 10.5–29.5)^(25)^ and nurse-led app-based home exercise program conducted by nurses, app developers, and physical therapists in 3 months (MD: 14.9; 95% CI: 7.82–21.98)^(36)^ demonstrated greatest MDs in range of motion. Further, the nurse-led app-based home exercise program indicated MDs in the pain (MD: –2.93; 95% CI: –1.54 to –4.32)^(36)^.

### Meta-Analysis Results

**Figure 4:**
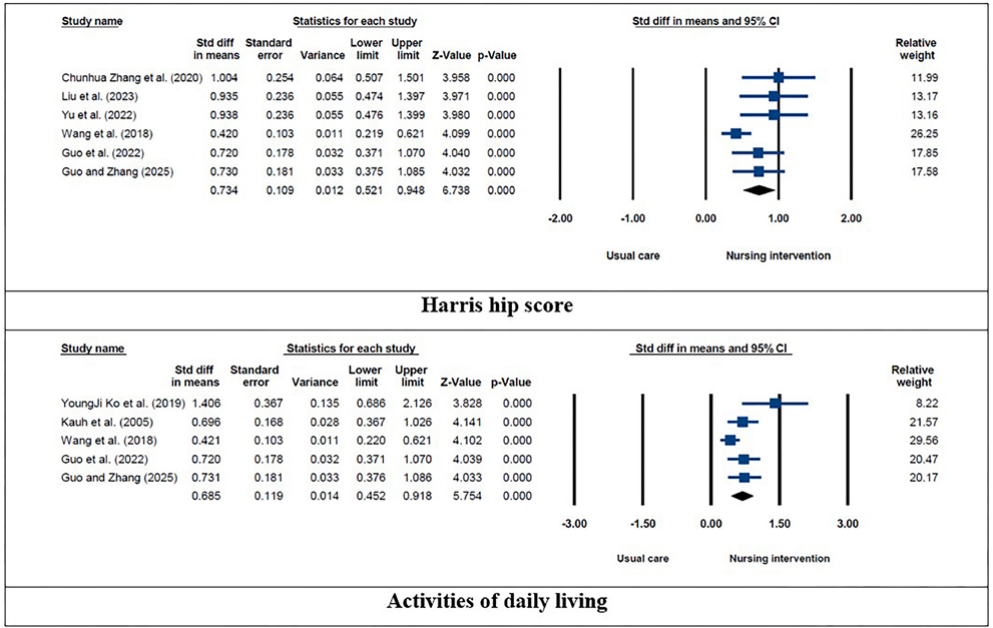
Meta-analysis conducted on standardized mean differences of rehabilitation outcomes after nursing interventions in patients with hip replacement.

**Figure 5:**
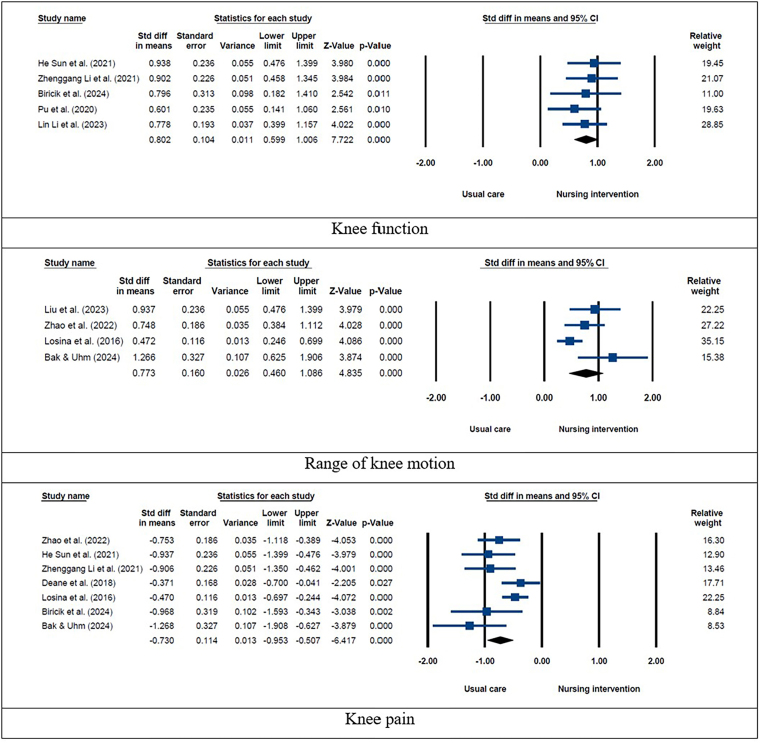
Meta-analysis conducted on standardized mean differences of rehabilitation outcomes after nursing interventions in patients with knee replacement.

Considering the significant variability in the outcomes, the meta-analysis was conducted employing a random effects model. Figures 4 and 5 illustrate the findings of the meta-analysis performed on the SMD of rehabilitation outcomes after nursing interventions in patients receiving hip and knee replacement. Among patients with hip replacements, the results indicated that nursing interventions compared with usual care can significantly increase Harris hip score (SMD: 0.734; 95% CI: 0.521–0.948; *P* < 0.001; I^2^ = 51.32) and activities of daily living (SMD: 0.685; 95% CI: 0.452–0.918; *P* < 0.001; I^2^ = 56.43). Among patients with knee replacements, the results showed that nursing interventions compared to usual care can significantly increase range of motion (SMD: 0.773; 95% CI: 0.460–1.086; *P* < 0.001; I^2^ = 60.98) and knee function (SMD: 0.802; 95% CI: 0.599–1.006; *P* < 0.001; I^2^ = 0.001) and decrease knee pain (SMD: –0.730; 95% CI: –0.953 to –0.507; *P* < 0.001; I^2^ = 53.17).

Table 3 describes GRADE profiles associated with the effect of nursing interventions on the rehabilitation of patients after knee and hip replacements. The assessment revealed that certainty related to the outcome of the Harris hip score was low, and that of activities of daily living, knee function, range of motion, and knee pain was moderate.

**Table 3:** GRADE profiles related to the effect of nursing interventions on the rehabilitation of patients after knee and hip replacements – Huai’an, JS, China, 2024.

Outcomes	Risk of bias	Inconsistency	Indirectness	Imprecision	Publication bias	Number (intervention/control)	SMD (95%CI)	Certainty
Harries hip score	Not serious	Not serious	Serious^a^	Not serious	Publication bias is strongly suspected^b^	442/441	0.734 (95% CI: 0.521–0.948)	⨁⨁◯◯ Low
Activities of daily living	Not serious	Not serious	Not serious	Not serious	Publication bias is strongly suspected^b^	421/419	0.685 (95% CI: 0.452–0.918)	⨁⨁⨁◯ Moderate
Knee function	Not serious	Not serious	Serious^a^	Not serious	None	201/200	0.802 (95% CI: 0.599–1.006)	⨁⨁⨁◯ Moderate
Range of motion	Not serious	Not serious	Not serious	Not serious	Publication bias is strongly suspected^b^	279/278	0.773 (95% CI: 0.460–1.086)	⨁⨁⨁◯ Moderate
Knee pain	Not serious	Not serious	Not serious	Not serious	Publication bias is strongly suspected^b^	416/415	−0.730 (95% CI: −0.953 to −0.507)	⨁⨁⨁◯ Moderate

^a^Downgraded for indirectness in the country.

^b^Publication bias was detected through Egger and Begg’s test (*p* < 0.05).

## DISCUSSION

Most studies (81%) included in this review were conducted after 2020. This indicates that this issue is an interesting topic, which has attracted the attention of researchers in recent years. Most studies (73.7%) used RCT as a valid study design for assessing this effectiveness. The quality assessment demonstrated that most of the reviewed papers have proper quality.

Various nursing interventions were used in the reviewed studies for the rehabilitation of patients undergoing knee and hip replacements, comprising clinical nursing, evidence-based interventions, nurse-led programs, remote nursing, self-care, painless rehabilitation nursing, perioperative nursing care, skilled nursing, individualized nursing, progressive nursing, CNC, predictive nursing, and nursing care in combination with other methods. Further, the time duration of the interventions was different in these studies from 7 days to 12 months. Of 19 reviewed studies, 17 showed that nursing interventions can improve the rehabilitation of patients after knee and hip replacement operations. Only two studies on remote nursing programs showed no significant associations between nursing interventions and rehabilitation in patients with knee replacement. The effectiveness of various nursing interventions was different.

Among the nursing interventions for patients with hip replacements, elastic stretch traction belt and pain assessment nursing care provided by nurses and medical staff in 6 months^(3)^, predictive nursing conducted by nurses and orthopedic surgical teams in 3 months^(38)^, and CNC performed by nurses, physicians, and rehabilitation therapists in 3 months^(37)^ had highest MDs in harries hip score, respectively. Further, predictive nursing showed the greatest MDs in activities of daily living^(38)^.

Yu et al. combined nursing care with the use of an elastic traction band to improve the rehabilitation of patients with hip replacement^(3)^. A team, comprising nurses and medical staff, performed this intervention for 6 months. It significantly affected the HHS among patients. Conventional rehabilitation training, including health education and medical guidance, supports postoperative recovery, but this often causes suboptimal results^(40)^. Studies indicate that the application of a self-made elastic traction belt combined with standardized nursing care during rehabilitation training significantly affects rehabilitation outcomes among patients after hip arthroplasty^(3)^.

Guo et al. utilized predictive nursing interventions for the rehabilitation of elderly patients. Nurses and orthopedic surgical teams conducted this intervention^(38)^. It significantly improved the HHS and activities of daily living of patients^(38)^. The predictive nursing strategy employs an evidence-based methodology to predict the needs of patients and prevent complications through early potential risk identification during the care process^(41)^. This approach encompasses comprehensive patient assessments, such as physical, psychological, and social aspects, and uses predictive analytics and tools to categorize patients according to their risk levels^(42)^. Such categorization enables the implementation of individualized interventions, continuous monitoring, and early clinical deterioration detection, thereby enhancing patient outcomes^(43)^. Over recent years, the significance of individualized care approaches has increased, especially in the context of complex medical procedures^(44,45)^.

Guo et al.^(37)^ also applied a CNC model on elderly patients receiving total hip arthroplasty^(37)^. Nurses, physicians, and rehabilitation therapists provided this intervention for 3 months. It significantly increased the HHS of patients. In CNC, patient health needs and available resources are assessed before hospital discharge. This approach includes health education, discharge instructions, telephone follow-ups, and home visits performed by a team of experienced practice nurses and various healthcare professionals^(37)^. Meanwhile, traditional nursing care primarily focuses on the perioperative phase alone, which exposes patients to several potential risks after discharge^(37)^.

Among the nursing interventions for patients undergoing knee replacements, perioperative nursing care conducted by orthopedic nurses during one month^(27)^ and increased recovery after surgery (ERAS) nursing combined with limb training performed by nurses and orthopedic physicians in 3 months^(28)^ exhibited the highest MDs for knee motion. Moreover, ERAS performed by a multidisciplinary team, comprising nurses, surgeons, anesthesiologists, nutritionists, physiotherapists, and pharmacists, in 12 months^(25)^ and a nurse-led app-based home exercise program conducted by nurses, app developers, and physical therapists in 3 months^(36)^ exhibited greatest MDs in range of motion. Further, nurse-led app-based home exercise programs indicated the highest MDs in pain^(36)^.

Sun et al.^(27)^ investigated the effect of perioperative nursing on knee function rehabilitation. This intervention was performed by orthopedic nurses for 1 month^(27)^. The efficacy of this intervention may originate from the involvement of specialized nurses^(27)^. Perioperative nursing emphasizes the crucial role of specialized nurses in the treatment process, which bolsters their sense of responsibility and identity within the nursing profession and contributes to the effectiveness of medical teams in improving treatment outcomes^(46)^. This intervention provides a foundation in evidence-based medicine that supports the advancement of nursing practices^(27)^.

Li et al. also combined ERAS nursing with limb training to increase the rehabilitation of knee function. A team of nurses and orthopedic physicians performed this intervention for 3 months^(28)^. In this study, limb exercise was added to the ERAS program as an evidence-based intervention, using a specialized team to enhance rehabilitation. ERAS integrates traditional nursing practices across all perioperative phases and optimizes these methods to achieve desired outcomes^(47)^. It ensures continuous postoperative recovery by establishing a specialized nursing team. This team receives systematic training in disease management and postoperative care techniques, which allows them to provide programmed and standardized care to patients pre- and postoperatively^(28)^. Moreover, early limb exercise reduces the risk of venous thrombosis, promotes the repair of adjacent tissues, and decreases joint injury-related pain^(28)^. The improvement in postoperative pain may be associated with the fact that post-surgery limb exercises improve knee joint ROM, increase muscle strength, and enhance joint endurance and coordination^(47)^. The results reveal that incorporating limb exercises within the ERAS framework can also improve knee functionality^(47)^.

Liu et al.^(25)^ investigated the effect of ERAS with multidisciplinary collaboration on nursing outcomes^(25)^. A multidisciplinary team, comprising surgeons, anesthesiologists, nurses, physiotherapists, nutritionists, and pharmacists, performed this intervention for 12 months. This intervention significantly increased the ROM in the patients^(25)^. The need for multidisciplinary collaboration in ERAS protocols is crucial, involving various healthcare professionals during the perioperative phase^(48)^. This strategy ensures that care is both standardized and tailored, grounded in the best current evidence, and aligned with unique patient needs^(49)^. Furthermore, interdisciplinary collaboration improves interaction and learning among healthcare providers, patients, and their families, resulting in consistent and high- quality care and increasing patient satisfaction and compliance^(50)^.

Bak and Uhm^(36)^ utilized a nurse-led app-based home exercise program for the rehabilitation of patients undergoing knee replacement and observed that this intervention exhibited a significant effect on ROM and knee pain of patients^(36)^. Nurses, app developers, and physical therapists performed this intervention for 3 months. Postoperative exercise is an integral part of the rehabilitation process aimed at restoring joint function. Research indicates that early exercises within the first week postoperatively result in favorable outcomes^(36)^. Mobile healthcare, which overcomes geographical and temporal limitations, is used for sharing health information and facilitating patient interactions with healthcare providers^(51,52)^. This method was successfully employed for the rehabilitation of patients after knee replacement. However, notably, this training must be based on a nurse-led program for obtaining suitable outcomes. In this approach, nurses play a pivotal role by providing comprehensive care, advocacy, and leadership. Nurses have distinct abilities and perspectives that allow them to care for the entire people and form an essential association between compassionate care and evidence-based practices^(53)^. Scattered measures cannot be associated with significant successes. Thus, two studies on remote nursing using patient-directed self-management of pain and MI-based postoperative care navigation demonstrated no meaningful outcomes^(10,39)^.

Altogether, the results of these studies indicate the importance of specialized nursing services in combination with other rehabilitation services among patients undergoing knee and hip replacement, which may be because knee and hip replacements require special attention. Hence, even after discharge, exercises and interventions need to continue under the supervision of a specialized team until the patient regains adequate functional ability.

Based on the assessment, certainty associated with the outcome of the HHS was low, and certainty related to the outcomes of activities of daily living, knee function, ROM, and knee pain was moderate. Further research is very likely to exhibit an important effect on our confidence in the estimate of effect and is likely to change the estimate for the outcomes with low certainty.

As a limitation, the design of intervention programs differed among different studies, which may also introduce some biases. Further, the follow-up time was different among studies. Moreover, differences between different genders and ages have not been investigated in the reviewed studies. As another limitation, non-English papers were not included in the review.

## CONCLUSION

Most reviewed studies revealed that nursing interventions can improve the rehabilitation of patients after knee and hip replacement. The effectiveness of various nursing interventions varied. The nursing interventions with the highest effectiveness included predictive nursing, continuous nursing, pain assessment nursing combined with an elastic stretch traction belt, perioperative nursing, ERAS nursing in combination with limb training, and a nurse-led app-based home exercise program. In summary, evidence indicates that individualized care and evidence-based strategies in combination with various exercises and training (e.g., rehabilitation exercises, postoperative limb exercises, limb exercises within the ERAS framework, early exercises within the first-week postoperative), which are provided by a multidisciplinary team with the leadership of a nurse, can significantly affect the physical rehabilitation among patients undergoing knee and hip replacement. Further, these interventions can be more successful when performed as continuous plans from pre- to postoperation and after discharge. Mobile apps, telephones, face-to-face visits, and other communication methods can be employed to continue these measures. Therefore, a novel model of nursing programs is developed based on comprehensive and continuous measures with an interactive approach combined with remote technology in the next studies. The effectiveness of novel nursing interventions compared with previous nursing interventions is supposed to be assessed in the next studies. Further, the effectiveness of these interventions is compared between various groups, in terms of age, gender, and other demographic characteristics.

## Data Availability

The data used to derive the findings in this study are available from the corresponding author upon reasonable request.

## SUPPLEMENTARY MATERIAL

The following online material is available for this article:

Table S1 – The search strategy in the present study – Huai’an, JS, China, 2024.
