# Supplementary material for: Effect of nursing interventions on the rehabilitation of patients after knee and hip replacements
Source: Rev Esc Enferm USP. 2025 Sep 15;59:e20250184. doi: 10.1590/1980-220X-REEUSP-2025-0184en (PMC12439537; doi:10.1590/1980-220X-REEUSP-2025-0184en)
Supplement: Supplementary file 1 [file 1980-220X-reeusp-59-e20250184-suppl1.pdf]

Rev Esc Enferm USP
https://doi.org/10.1590/1980-220X-REEUSP-2025-0184en

Supplementary Material to “Effect of nursing interventions on the rehabilitation of patients after knee and hip replacements”

Table S1 – The search strategy in the present study - Huai’an, JS, China, 2024.

Database	Search strategy
PubMed	((((((((("Nursing Care"[MeSH Terms] OR "Nursing Process"[MeSH Terms] OR "Perioperative Nursing"[MeSH Terms] OR "Postoperative Care"[MeSH Terms] OR "nurse-led"[Title/Abstract] OR "care intervention*"[Title/Abstract] OR "nursing management"[Title/Abstract] OR "continuous nursing"[Title/Abstract]))))))))) AND (((((((((((((((("Arthroplasty, Replacement, Knee"[MeSH Terms] OR "Arthroplasty, Replacement, Hip"[MeSH Terms] OR "Knee Prosthesis"[MeSH Terms] OR "Hip Prosthesis"[MeSH Terms] OR "knee replacement"[Title/Abstract] OR "hip replacement"[Title/Abstract] OR "total knee arthroplasty"[Title/Abstract] OR TKA[Title/Abstract] OR "total hip arthroplasty"[Title/Abstract] OR THA[Title/Abstract] OR "joint replacement"[Title/Abstract] OR "knee surgery"[Title/Abstract] OR "hip surgery"[Title/Abstract] OR "prosthetic knee"[Title/Abstract] OR "prosthetic hip"[Title/Abstract])))))))))))))))) AND ((((((((((((((((("Rehabilitation"[MeSH Terms] OR "Rehabilitation Nursing"[MeSH Terms] OR "Recovery of Function"[MeSH Terms] OR "Physical Therapy Modalities"[MeSH Terms] OR rehab*[Title/Abstract] OR "functional recovery"[Title/Abstract] OR "physical rehabilitation"[Title/Abstract] OR "mobility"[Title/Abstract] OR "range of motion"[Title/Abstract] OR ROM[Title/Abstract] OR "functional outcome*"[Title/Abstract] OR "postoperative recovery"[Title/Abstract] OR "physical function"[Title/Abstract] OR "quality of life"[Title/Abstract] OR QoL[Title/Abstract] OR "exercise therapy"[Title/Abstract])))))))))))))))))
ISI	((TS=(nurs* OR "nurse-led" OR "care intervention*" OR "nursing management" OR "nursing support" OR "continuous nursing" OR "nonpharmacologic intervention*")) AND (TS=("knee replacement" OR "hip replacement" OR "total knee arthroplasty" OR TKA OR "total hip arthroplasty" OR THA OR "joint replacement" OR "knee surgery" OR "hip surgery" OR

	"prosthetic knee" OR "prosthetic hip")) AND (TS=(rehab* OR "functional recovery" OR "physical rehabilitation" OR "mobility" OR "range of motion" OR ROM OR "functional outcome*" OR "postoperative recovery" OR "physical function" OR "quality of life" OR QoL OR "exercise therapy")))
SCOPUS	((TITLE-ABS-KEY (nurs* OR "nurse-led" OR "care intervention*" OR "nursing management" OR "nursing support" OR "nursing care" OR "continuous nursing" OR "nonpharmacologic intervention*") AND (TITLE-ABS-KEY ("knee replacement" OR "hip replacement" OR "total knee arthroplasty" OR TKA OR "total hip arthroplasty" OR THA OR "joint replacement" OR "knee surgery" OR "hip surgery" OR "prosthetic knee" OR "prosthetic hip") AND (TITLE-ABS-KEY (rehab* OR "functional recovery" OR "physical rehabilitation" OR "mobility" OR "range of motion" OR ROM OR "functional outcome*" OR "postoperative recovery" OR "physical function" OR "quality of life" OR QoL OR "exercise therapy"))
