# Supplementary material for: Effect of nursing interventions on the rehabilitation of patients after knee and hip replacements
Source: Rev Esc Enferm USP. 2025 Sep 15;59:e20250184. doi: 10.1590/1980-220X-REEUSP-2025-0184en (PMC12439537; doi:10.1590/1980-220X-REEUSP-2025-0184en)
Supplement: Translation

# Translation: Efecto de las intervenciones de enfermería en la rehabilitación de pacientes después de reemplazos de rodilla y cadera

**DOI:** 10.1590/1980-220X-REEUSP-2025-0184es

**Authors:** Qing Sheng, Guangling Wang, Pengfei Wang

**Affiliations:** 1 Jiangsu Province Hospital, The Third Ward of the Department of Orthopedics, Nanjing, China.; 2 Huai’an Economic and Technological Development Zone Hospital, Department of Orthopedics, Huai’an, Jiangsu, China.

**Keywords:** Atención de Enfermería, Rehabilitación, Paciente, Prótesis de la Rodilla, Prótesis de Cadera

## Abstract

**Objetivo:**

Esta revisión sistemática y metaanálisis tuvo como objetivo evaluar el efecto de las intervenciones de enfermería, considerando su importancia en la rehabilitación de pacientes sometidos a reemplazo de rodilla y cadera.

**Métodos:**

Se realizaron búsquedas sistemáticas en tres bases de datos. Dos investigadores revisaron cuidadosamente los textos completos de los estudios seleccionados y extrajeron los datos necesarios. Posteriormente, se realizó un metaanálisis.

**Resultados:**

De los 19 estudios revisados, 17 revelaron que las intervenciones de enfermería pueden mejorar la rehabilitación de los pacientes tras cirugías de reemplazo de rodilla y cadera. En pacientes con reemplazo de cadera, los resultados indicaron que las intervenciones de enfermería pueden aumentar significativamente la puntuación de Harris en la escala de cadera y las actividades de la vida diaria, en comparación con la atención habitual. En pacientes con reemplazo de rodilla, los resultados revelaron que las intervenciones de enfermería pueden mejorar significativamente el rango de movimiento y la función de la rodilla, así como reducir el dolor de rodilla, en comparación con la atención habitual.

**Conclusiones:**

La evidencia revela que la atención individualizada y las estrategias basadas en la evidencia, combinadas con diversos ejercicios y entrenamiento proporcionados por un equipo multidisciplinario, influyen significativamente en la rehabilitación física de los pacientes sometidos a reemplazo de rodilla y cadera.

## INTRODUCCIÓN

La cirugía de prótesis de rodilla consiste en sustituir las superficies articulares desgastadas de la articulación de la rodilla por implantes artificiales para aliviar el dolor y restaurar la función articular^(1)^. Esta técnica quirúrgica puede ayudar en el tratamiento de enfermedades, como la artrosis de rodilla, la artritis reumatoide y la artritis traumática^(2)^. Además, la cirugía de prótesis de cadera sirve como método de tratamiento para algunas enfermedades, como la necrosis avascular de la cabeza femoral y las fracturas del cuello femoral^(3)^. Esta técnica es una opción de tratamiento popular y eficaz para diversos trastornos de la cadera, y se utiliza predominantemente en entornos médicos^(4)^. Así, estos procedimientos quirúrgicos reducen significativamente las molestias y mejoran la calidad de vida de los pacientes^(5)^.

Apesar de que las prótesis de rodilla y cadera ofrecen ventajas considerables, también pueden asociarse a riesgos y posibles complicaciones, como traumatismos importantes, pérdida de sangre y molestias^(6,7)^. En consecuencia, estos procedimientos pueden causar dolor postoperatorio, limitación de la amplitud de movimiento, disminución de la velocidad de la marcha, disminución de la fuerza muscular y las capacidades físicas, deterioro de la capacidad para subir escaleras y aumento del riesgo de caídas. Estos problemas afectan a la reanudación de las actividades cotidianas y a la calidad de vida de los pacientes^(8)^.

Por lo tanto, no se puede ignorar la importancia de la rehabilitación postoperatoria, aunque las cirugías pueden acelerar la vuelta a las actividades cotidianas^(9)^. Los programas de rehabilitación temprana afectan a los resultados funcionales a largo plazo y a la calidad de vida de los pacientes^(10)^. Pruebas recientes han puesto de relieve la importancia clínica de las intervenciones de enfermería como programas de rehabilitación^(11,12)^. Dichas intervenciones mejoran el éxito de los resultados quirúrgicos, optimizan el proceso de tratamiento, reducen las complicaciones postoperatorias y mejoran la calidad de vida de los pacientes^(13–15)^.

Existen diversas intervenciones de enfermería para la rehabilitación de los pacientes sometidos a intervenciones quirúrgicas. Estas intervenciones de enfermería se han diseñado basándose en diferentes enfoques. Por ejemplo, los cuidados de enfermería centrados en el paciente tienen en cuenta las necesidades únicas de los pacientes a la hora de tomar decisiones^(16)^. En cambio, la teoría del déficit de autocuidados se centra en la capacidad de autocuidado de un individuo^(17)^. Las prácticas de enfermería integrales, individualizadas y de alta calidad contribuyen a los cuidados centrados en el paciente. Las estrategias, como los programas dirigidos por enfermeras, la asistencia telefónica y los planes educativos, se asocian a la teoría del déficit de autocuidados. Por lo tanto, las diferentes intervenciones de enfermería pueden estar relacionadas con diversas eficacias en la rehabilitación de los pacientes.

Teniendo en cuenta la importancia de las intervenciones de enfermería en la rehabilitación de los pacientes sometidos a prótesis de rodilla y cadera, es necesario especificar la eficacia de las distintas intervenciones de enfermería. De este modo, se identifican intervenciones de enfermería útiles para cada uno de los resultados de la rehabilitación. Por lo tanto, se justifica un estudio exhaustivo que resuma los resultados de estudios anteriores. Zhang et al.^(18)^, en una revisión sistemática y un metaanálisis, investigaron la eficacia de la mejora de la recuperación postoperatoria en los cuidados perioperatorios de enfermería entre pacientes ancianos con artroplastia de cadera y rodilla^(18)^. Además, Wang et al.^(19)^, en una revisión sistemática y un metaanálisis, evaluaron el efecto de la intervención preoperatoria con ejercicios en la rehabilitación tras una artroplastia total de rodilla^(19)^. Sin embargo, los procedimientos y la eficacia de las intervenciones postoperatorias varían en comparación con las intervenciones preoperatorias. Por lo tanto, es necesario estudiar exhaustivamente el efecto de las intervenciones postoperatorias. Moon et al., en una revisión sistemática y un metaanálisis, investigaron la eficacia de las intervenciones postoperatorias en pacientes con sustitución total de rodilla/cadera, pero sólo se centraron en los efectos de las intervenciones de tratamiento del dolor dirigidas por enfermeras^(20)^. Mientras tanto, es necesario tener en cuenta otros tipos de enfermería postoperatoria. Por lo tanto, para abordar estas brechas de conocimiento, la presente revisión sistemática y metaanálisis tuvo como objetivo evaluar el efecto de diversas intervenciones de enfermería en la rehabilitación física de los pacientes tras la sustitución de rodilla y cadera.

## MÉTODO

Este estudio de revisión y metanálisis evaluó la efectividad de diversas intervenciones físicas de enfermería en el proceso de recuperación de las personas sometidas a cirugías de reemplazo de rodilla y cadera. Por lo tanto, la pregunta de investigación fue «si las intervenciones de enfermería pueden afectar la rehabilitación de los pacientes después de los reemplazos de rodilla y cadera». La presente revisión siguió el protocolo del Instituto Joanna Briggs (JBI). Para redactar el artículo se utilizó la directriz de los Elementos de Información Preferidos para Revisiones Sistemáticas y Metaanálisis (PRISMA)^(21)^. Además, esta revisión exhaustiva y metaanálisis está formalmente documentada en el registro PROSPERO (CRD420251039484).

### Criterios de Elegibilidad

En esta investigación, los autores no tuvieron en cuenta restricciones asociadas al origen étnico, la nacionalidad, el sexo, la edad o factores comparables. Los criterios de inclusión comprendieron una amplia gama de artículos publicados en inglés. Además, como criterios de exclusión, se excluyeron los artículos de conferencias, notas, revisiones de conferencias, capítulos de libros, editoriales, encuestas breves, cartas y artículos de revisión. Además, se eliminaron los estudios de calidad insuficiente.

### Estrategia de Búsqueda

Para alcanzar los objetivos de este estudio, se realizó una búsqueda exhaustiva y metódica en Web of Science, Scopus y PubMed para encontrar artículos relevantes publicados hasta el 24 de marzo de 2025. Se identificaron las palabras clave iniciales para realizar una búsqueda sistemática. A continuación, se clasificaron los artículos en función de sus similitudes semánticas y conceptuales (Tabla 1). La Tabla 1 representa la estrategia de búsqueda empleada en el presente estudio.

**Tabla 1:** Categorización de palabras clave basada en similitudes semánticas y conceptuales – Huai’an, JS, China, 2024.

Categorías	Palabras llave relacionadas
Cuidados e intervenciones de enfermería	Nursing care, nursing process, perioperative nursing, postoperative care, nurse-led, care intervention, nursing management, continuous nursing, care intervention, nursing support, nonpharmacologic intervention
Procedimientos quirúrgicos e implantes	“Arthroplasty, replacement, knee,” “arthroplasty, replacement, hip,” “knee prosthesis,” “hip prosthesis,” “knee replacement,” “hip replacement,” “total knee arthroplasty,” “total hip arthroplasty,” “joint replacement,” “knee surgery,” “hip surgery,” “prosthetic knee,” “prosthetic hip”
Rehabilitación y recuperación	Rehabilitation,” “rehabilitation nursing,” “recovery of function,” “physical therapy modalities,” “functional recovery,” “physical rehabilitation,” “mobility,” “range of motion,” “functional outcome,” “postoperative recovery,” “physical function,” “quality of life,” “exercise therapy”

Estas palabras clave se combinaron sistemáticamente mediante operadores booleanos para establecer una estrategia de búsqueda a medida. A continuación, esta estrategia se aplicó en todas las bases de datos para recolectar la bibliografía pertinente.

### Selección de Estudios

Todos los artículos recuperados se importaron a EndNote. Posteriormente se identificaron y eliminaron los duplicados. Dos investigadores (Q.S. y P.W.) examinaron de forma independiente los artículos restantes evaluando sus títulos y resúmenes. A continuación, se eliminaron los estudios irrelevantes según los criterios de inclusión y exclusión. Además, ambos investigadores (Q.S. y P.W.) revisaron las referencias de los artículos seleccionados para descubrir cualquier estudio pertinente. Posteriormente, se evaluaron los textos completos de los artículos para determinar los trabajos pertinentes.

### Evaluación de la Calidad

La calidad de los estudios seleccionados se evaluó utilizando las herramientas de valoración crítica del JBI diseñadas para ensayos clínicos randomizados (ECR), estudios de cohortes, de casos y controles y cuasiexperimentales. La lista de verificación del JBI está bien considerada para evaluar el rigor metodológico de los estudios^(22)^. Los criterios del JBI para la evaluación de los ECR incluían el uso de una verdadera aleatorización utilizada para la asignación de los grupos de participantes, la ocultación de la asignación de los grupos de tratamiento, la similitud de los grupos de tratamiento al inicio del estudio, participantes cegados a la asignación del tratamiento, información de las personas que proporcionan el tratamiento cegadas a la asignación del tratamiento, similitud del tratamiento en grupos administrados distintos de la intervención de interés, evaluadores de resultados cegados a la asignación del tratamiento, similitud en la medición de los resultados para los grupos de tratamiento, medición de los resultados con un método fiable, finalización del seguimiento, análisis de los participantes en los grupos a los que fueron asignados aleatoriamente, adecuación del análisis estadístico, y adecuación del diseño del ensayo y consideración de cualquier desviación del diseño estándar del ECR en el análisis. Se emplearon los criterios del JBI para evaluar los estudios de cohortes utilizando los criterios: similitud entre dos grupos, similitud de la medición de la exposición, validez y fiabilidad de la medición de la exposición, identificación de factores de confusión, estrategias para abordar los factores de confusión, información de los participantes sobre el resultado al inicio del estudio, validez y fiabilidad de la medición de los resultados, suficiencia del tiempo de seguimiento, finalización del seguimiento, estrategias para abordar el seguimiento incompleto y adecuación del análisis estadístico. Los estudios de casos y controles se evaluaron utilizando los criterios: comparabilidad de los grupos de casos y controles, emparejamiento adecuado de los grupos de casos y controles, similitud de los criterios para identificar los grupos de casos y controles, medición de la exposición con un método estándar, válido y fiable, evaluación de la exposición con un método similar para los grupos de casos y controles, identificación de los factores de confusión, estrategias para tratar los factores de confusión, adecuación del periodo de exposición y adecuación del análisis estadístico. Los estudios semiexperimentales se evaluaron utilizando los criterios: claridad de causa y efecto, existencia de un grupo de control, comparaciones similares entre los participantes, exposición similar entre los participantes, mediciones múltiples de los resultados, comparaciones similares de los resultados de los participantes, medición fiable de los resultados, estado del seguimiento y adecuación del análisis estadístico. Se completó cada lista de comprobación y se sumaron las respuestas positivas para calcular una puntuación total. Según estas puntuaciones, los estudios se clasificaron en tres grupos de calidad: baja, moderada y alta.

### Extracción de Datos

Dos autores (Q.S. y P.W.) realizaron la tarea de forma independiente utilizando una plantilla estandarizada para garantizar la minuciosidad y precisión del proceso de extracción de datos. Esta plantilla se desarrolló para recolectar una amplia gama de detalles, incluidos el autor(es) y el año de publicación, el país, el diseño del estudio, el tamaño de la muestra, el sexo, la edad, el tipo de intervención de enfermería, la duración de la intervención de enfermería, los proveedores de servicios, el método de evaluación de resultados, la región corporal, los resultados y los desenlaces. Este paso garantizó que toda la información recopilada fuera precisa, completa y se ajustara a los criterios predefinidos.

### Síntesis y Análisis de los Datos

En esta investigación, la concordancia entre los hallazgos de dos investigadores (Q.S. y P.W.) se evaluó mediante la prueba kappa de Cohen^(23)^. En el presente estudio se obtuvo un valor de 0,91, que resultó aceptable. Además de los resultados descriptivos, se realizó un metaanálisis de los valores de las diferencias de medias estandarizadas (DME) asociadas a diversos resultados de rehabilitación comunicados en algunos estudios. Para el análisis de los datos se utilizó la tercera versión del programa informático de metaanálisis exhaustivo. Además, se utilizó el programa informático GRADE pro para evaluar la certeza de los resultados.

## RESULTADOS

### Resultados de la Búsqueda y Selección de Estudios

Las bases de datos proporcionaron un total de 2090 artículos. De éstos, se eliminaron 407 artículos duplicados en el programa informático EndNote, lo que dio como resultado 1,683 artículos disponibles para el cribado. Aplicando los criterios de exclusión, se eliminaron 240 estudios, que incluían 29 ponencias de congresos, 12 informes de casos y 199 artículos de revisión, lo que dejó 1,443 artículos para su posterior evaluación. De éstos, se excluyeron 1,400 estudios irrelevantes tras el proceso de cribado de títulos y resúmenes. Finalmente, quedaron 43 artículos para la revisión del texto completo, de los cuales 19 se incluyeron en el estudio. Estos trabajos se realizaron entre 2004 y 2025, la mayoría después de 2020 (14 [73,6%] estudios). La Figura 1 ilustra el Flujograma de PRISMA.

**Figura 1:** Diagrama de flujo PRISMA.

### Especificación de los Artículos

La Tabla 2 muestra los datos totales de los artículos revisados. La Figura 2 ilustra la distribución estadística de los estudios revisados según diversas características. El diseño de los estudios revisados fue ECR (14 estudios), cohorte (2 estudios), caso-control (1 estudio) y cuasiexperimental (2 estudios). Estos estudios se realizaron en China (12 estudios), Corea del Sur (2 estudios), Estados Unidos (2 estudios), Reino Unido (1 estudio), Taiwán (1 estudio) y Turquía (1 estudio). Los estudios se realizaron en varones (2 estudios), mujeres (1 estudio) y ambos sexos (16 estudios). La edad media de los participantes oscila entre 38,40 y 80,60 años. Estos estudios incluyeron 2234 participantes.

**Tabla 2:** Detalles de los estudios revisados – Huai’an, JS, China, 2024.

Autor (año)	país	Tipo de estudio	Tamaño de muestra	Género	Media de edad (años)	Tipo de intervención de enfermería	Duración de la intervención de enfermería (meses)	Provisión de servicios	Método de evaluación de resultados	Región	Resultados	Consecuencias	Calidad
Zhang and Xiao (2020)^(24)^	China	Ensayo clínico randomizado (ECR)	70	Ambos	59–60 años	Camino clínico de enfermería	2–3 semanas	Equipo multidisciplinar (enfermeras, fisioterapeutas y cirujanos)	Puntuación Harris de cadera (*Harris Hip Score* -HHS) y puntuación de satisfacción	Cadera	La intervención mejoró la función de la cadera, acortó el tiempo de recuperación y aumentó la satisfacción.	HHS: Diferencia de medias (DM): 7,05 (intervalo de confianza [IC] del 95%: 3,70–10,40) Satisfacción: DM: 2,97 (IC 95%: 1,56–4,38)	Q2
Liu et al. (2023)^(25)^	China	Estudio de cohortes retrospectivo	80	Hombres	64,7 años	Recuperación postoperatoria mejorada (Enhanced recovery after surgery - ERAS) con medidas multidisciplinares	–	Equipo multidisciplinar (cirujanos, anestesistas, enfermeros, fisioterapeutas, nutricionistas y farmacéuticos)	HHS, amplitud de movimiento (*Range of Motion-* ROM) y complicaciones	Cadera y rodilla	La intervención tuvo un efecto positivo en los resultados de la rehabilitación, incluida la recuperación funcional.	Complicaciones postoperatorias: Sin diferencias significativas (cociente de riesgos [CR] ≈ 1, *p* > 0,05). ROM: MD: 20 (95% IC: 10,5–29,5) HHS: MD: 5 (95% IC: 2,62–7,38)	Q2
Yu et al. (2024)^(3)^	China	Ensayo clínico prospectivo comparativo	80	Ambos	73,0 años	Cinturón elástico de tracción y cuidados de enfermería para la evaluación del dolor	6 meses	Enfermeras y personal médico	Escala HHS y SER (autoeficacia)	Cadera	Mejora de los resultados de la rehabilitación en la función de la cadera, la autoeficacia y la calidad de vida.	HHS: MD: 16,09 (95% IC: 8,45–23,73) Autoeficacia: MD: 8,22 (95% IC: 4,32–12,12)	Q1
Zhao et al. (2022)^(26)^	China	ECR	124	Ambos	74,29	Enfermería de rehabilitación sin dolor	6 meses	Enfermeros	Circunferencia de la rodilla, dolor de rodilla, función de la rodilla, agitación y duración del sueño	Rodilla	El grupo de observación demostró una reducción del dolor, una mejora de la función de la rodilla y un menor índice de agitación en comparación con el grupo de control.	Dolor en Rodilla: MD: −1,25 (95% IC: 0,66–1,84) Movimientos en Rodilla: MD: 12,06 (95% IC: 6,33–17,79)	Q3
Sun et al. (2021)^(27)^	China	ECR	80	Ambos	38,48	Cuidados perioperatorios de enfermería	1 mes	Enfermeros ortopedistas	Escala de función de la articulación de la rodilla de Harris y escala analógica visual (EVA)	Rodilla	Mejora significativa en las puntuaciones de dolor y satisfacción de enfermería en el grupo de estudio en comparación con el grupo de control.	Puntuación de la función de la rodilla: MD: 10,3 (95% IC: 5,41–15,19) Puntuación del dolor: DM: –1,8 (95% IC: 0,95–2,66)	Q1
Chang et al. (2024)^(8)^	Taiwan	ECR	52	Ambos	73,44	Programa de enseñanza híbrido dirigido por enfermeros	4 meses	Enfermeros y fisioterapeutas	Fuerza del cuádriceps y puntuación de lesiones de rodilla y osteoartritis (KOOS)	Rodilla	Un programa de enseñanza híbrido mejoró los resultados de la rehabilitación, incluida la fuerza muscular y la función de la rodilla.	Fuerza del cuádriceps: MD: 5,2 (95% IC: 2,73–7,67) Puntuación KOOS: MD: 12,4 (95% IC: 6,51–18,29)	Q2
Li et al. (2021)^(28)^	China	ECR	86	Ambos	–	Enfermería ERAS combinada con formación en extremidades	3 meses	Enfermeros y médicos ortopédicos	EVA, puntuación de Judet y escala del Instituto Nacional de Salud del ACV	Rodilla	La enfermería ERAS combinada con el entrenamiento de las extremidades mejora los resultados de la rehabilitación.	Puntuación de dolor: DM: –1,5 (95% IC: 0,79–2,21) Función de la articulación de Rodilla: DM: 8,2 (IC 95%: 4,30–12,10) Función neurológica: DM: 2,3 (95% IC: 1,21–3,39)	Q1
Ko et al. (2019)^(29)^	Corea del Sur	Estudio cuasiexperimental	37	Ambos	–	Programa de cuidados transitorios individualizados (ITCP)	–	Enfermeros	Actividades de la vida diaria y puntuaciones cronometradas de levantarse	Caderas	El ITCP es eficaz para reducir el deterioro funcional en adultos mayores tras una artroplastia de cadera.	Deterioro funcional (DF): MD: 2,2 (95% IC: 1,15–3,25) Actividades de la vida diaria (AVD): MD: 4,4 (95% IC: 2,31–6,49)	Q1
Deane et al. (2018)^(39)^	Reino Unido	ECR	144	Hombres	69,8	Autogestión del dolor dirigida por el paciente	–	Las enfermeras y el equipo de investigación	EVA para el dolor	Rodilla	La automedicación no condujo a un mejor control del dolor en comparación con los pacientes atendidos por enfermeras.	Dolor: MD: −0,99 (95% IC: 0,11–1,87).	Q1
Kauh et al. (2005)^(30)^	Estados Unidos	Estudio piloto observacional retrospectivo	150	Mujeres	80,6	Enfermería especializada en rehabilitación geriátrica	1 año	Enfermeras geriátricas y un equipo interdisciplinar	Medida de los resultados de la rehabilitación ROM para las actividades de la vida diaria y la movilidad	Cadera	Esta estrategia demostró ser eficaz para mejorar los resultados de los pacientes y minimizar el uso innecesario de servicios de atención de salud tras una enfermedad grave.	Actividades de la vida diaria: MD: 0,41 (95% IC: 0,22–0,60) Movilidad: MD: 0,29 (95% IC: 0,15–0,43)	Q1
Losina et al. (2016)^(10)^	Estados Unidos	ECR	308	Ambos	66,0	Atención posoperatoria basada en entrevistas motivacionales (EM)	6 meses (10 calls)	Cirujano, enfermero a domicilio y fisioterapeuta	WOMAC pain score, satisfaction, and ROM	Rodilla	Los participantes del grupo de intervención con navegación no mostraron una mejora funcional más significativa que los del grupo de control.	Pain: MD: –3 (95% IC: −7,00–1,00) Satisfaction: MD: 8,00 (95% IC: 4,2–11,8) ROM: MD: 2,00 (95% IC: 1,05–2,95)	Q1
Biricik et al. (2024)^(31)^	Turquía	ECR	44	Ambos	66,3	Educación del paciente asistida por vídeo (EPV) sobre cuidados postoperatorios, AVD y ejercicios.	3 meses	Enfermeros (educadores primarios) y fisioterapeutas	Puntuación WOMAC y SF-36	Rodilla	Esta intervención mejoró la función de la rodilla, aumentó la calidad de vida y redujo las complicaciones posteriores a la artroplastia de rodilla.	Dolor: DM: –3,5 (IC 95%: –5,7 a –1,3) Función física: DM: –10,6 (IC 95%: –18,7 a –2,5) Rol físico: MD: 30,7 (95% IC: 7,4–54,0)	Q3
Wang et al. (2018)^(32)^	China	ECR	389	Ambos	55,67	Plataforma de atención ortopédica domiciliaria por Internet (WeChat) con apoyo de enfermeros especialistas (monitorización de la rehabilitación y programación de citas).	6 meses	Enfermeros ortopédicos	HHS, Índice Barthel (BI), y escala SF-36	Cadera	Esta intervención afectó significativamente la función articular, las actividades de la vida diaria y la calidad de vida en el grupo de intervención.	MD: HHS: MD: 6,07 (IC del 95%: 3,19–8,95) Actividades de la vida diaria (BI): 5,02 (IC del 95%: 2,64–7,40) Calidad de vida (SF–36): 6,78 (95% IC: 3,56–10,0)	Q1
Pua et al. (2020)^(33)^	China	ECR	76	Ambos	67,8	Enfermería individualizada (enfermería interactiva, control del dolor, ejercicios de rehabilitación, apoyo psicológico y prevención de complicaciones)	30 meses	Enfermeros clínicos	Puntuación del Hospital for Special Surgery (Cirugía Especial) (HSS)	Rodilla	Las intervenciones de enfermería individualizadas mejoraron la recuperación de la funcionalidad de las extremidades y la calidad de vida de los pacientes.	Puntuación funcional: MD: 1,8 (IC del 95%: 0,43–3,17)	Q3
Li et al. (2023)^(34)^	China	No-ECR	115 pacientes	Ambos	≥60	Enfermería progresiva	7 días	Enfermeros clínicos	Función de la rodilla y complicaciones	Rodilla	La enfermería progresiva mejoró la recuperación funcional, redujo las complicaciones y mejoró la calidad de vida.	Puntuación de la función de la rodilla: MD: 1,8 (IC del 95%: 0,95–2,66)	Q1
Xiao et al. (2024)^(35)^	China	Estudio retrospectivo de casos y controles.	90	Ambos	45,04	Control del dolor dirigido por enfermeras	–	Equipo multidisciplinar (enfermeras, anestesistas, médicos, rehabilitadores y farmacéuticos)	ROM, HHS, complicaciones y satisfacción (NSNS)	Cadera	Esta intervención mejoró el control del dolor, la satisfacción y la función articular, y redujo las complicaciones.	Eficacia de la intervención para el dolor: DM: 1,8 (IC del 95%: 0,94–2,66) ROM: DM: 13,22 (IC del 95%: 6,94–19,5) Complicaciones: DM: – 14,55 (IC del 95%: – 7,64 a −21,46) Satisfacción: DM: 17,07 (IC del 95%: 8,96–25,18)	Q2
Bak and Uhm (2024)^(36)^	Corea del Sur	Cuasi-experimental	45	Ambos	66,78	Programa de ejercicios en casa basado en una aplicación y dirigido por enfermeras (vídeos y notificaciones push).	3 meses	Enfermeras, desarrolladores de aplicaciones y fisioterapeutas.	EVA (dolor), ROM, escala de autoeficacia en el ejercicio y NSNS (satisfacción)	Rodilla	Los cuidados de enfermería mejoraron el control del dolor, la amplitud de movimiento, la autoeficacia y la satisfacción.	Dolor: DM: −2,93 (IC del 95%: −1,54 a −4,32) ROM: DM: 14,9 (IC del 95%: 7,82–21,98) Autoeficacia: DM: 2,81 (IC del 95%: 1,48–4,14)	Q2
Guo et al. (2022)^(37)^	China	ECR	134	Ambos	68,40	Cuidados de enfermería continuos (CNC)	3 meses	Enfermeras, médicos y terapeutas de rehabilitación.	Índice HHS y Barthel (AVD)	Cadera	La atención de enfermería continua mejoró la función de la cadera y las actividades de la vida diaria.	HHS: MD: 8,41 (IC del 95%: 4,42–12,40) Índice de Barthel (AVD): MD: 5,90 (IC del 95%: 3,10–8,70)	Q1
Guo and Zhang (2025)^(38)^	China	ECR	130	Ambos	70,5	Enfermería predictiva (basada en la evaluación de riesgos)	3 meses	Enfermeras y equipos quirúrgicos ortopédicos	HHS, índice de Barthel (AVD), complicaciones y puntuaciones de satisfacción.	Cadera	La atención de enfermería predictiva se asoció con estancias hospitalarias más cortas, mayores niveles de satisfacción, mejora de la función cadera, mejores puntuaciones en las actividades de la vida diaria y reducción de las complicaciones.	HHS: MD: 12,1 (IC del 95%: 6,35–17,85) Índice de Barthel (AVD): MD: 13,2 (IC del 95%: 6,93–19,47)	Q1

**Figura 2:** Distribución estadística de los estudios revisados en función de diversas características.

Las intervenciones de enfermería incluyeron enfermería clínica (1 estudio), intervenciones basadas en la evidencia (1 estudio), programas dirigidos por enfermeras (3 estudios), enfermería a distancia (3 estudios), autocuidados (1 estudio), enfermería de rehabilitación sin dolor (1 estudio), cuidados perioperatorios de enfermería (2 estudios), enfermería especializada (1 estudio), enfermería individualizada (2 estudios), enfermería progresiva, cuidados continuos de enfermería (Continuous Nursing Care - CNC) (1 estudio), enfermería predictiva (1 estudio) y cuidados de enfermería combinados con otros métodos (2 estudios). Además, 9 y 11 estudios se realizaron en pacientes con prótesis de cadera y rodilla, respectivamente. La duración de las intervenciones osciló entre 7 días y 30 meses. En estos estudios, los proveedores de servicios incluían enfermeros generales, enfermeros ortopédicas, enfermeros geriátricas, enfermeros de atención domiciliaria, enfermeros clínicas, fisioterapeutas, fisioterapeutas, terapeutas de rehabilitación, cirujanos, médicos ortopédicos, anestesistas, nutricionistas, farmacéuticos, rehabilitadores y otro personal de salud. Los resultados más predominantes de la rehabilitación fueron la puntuación Harris de cadera (*Harris Hip Score* - HHS), la amplitud de movimiento, la autoeficacia, la puntuación de dolor, la función de rodilla o cadera, la función neurológica, las actividades de la vida diaria, la movilidad, la calidad de vida, la puntuación de satisfacción y las complicaciones.

La Figura 3 ilustra la distribución estadística de los estudios revisados según el nivel de calidad. Según los resultados relativos a la evaluación de la calidad de los artículos, 11, 5 y 3 estudios se clasificaron en el grupo de artículos de calidad alta, moderada y baja, respectivamente. Los resultados revelaron que los niveles de calidad de los estudios revisados no podían afectar a las conclusiones del presente estudio, ya que sólo tres (15,8%) estudios eran de baja calidad.

**Figura 3:** Distribución estadística de los estudios revisados según su nivel de calidad.

### Resultados Principales

De los 19 estudios revisados, 17 demostraron que las intervenciones de enfermería pueden mejorar la rehabilitación de los pacientes después del reemplazo de rodilla y cadera^(3,8,24–38)^. Dos estudios mostraron asociaciones no significativas entre las intervenciones de enfermería y la rehabilitación en pacientes con reemplazo de rodilla^(10,39)^. El tipo de intervención de enfermería en estos dos estudios incluye la enfermería a distancia, que comprende el autocontrol del dolor dirigido por el paciente^(39)^ y la navegación de los cuidados postoperatorios basada en la entrevista motivacional (EM)^(10)^.

En los estudios se evaluaron diversos resultados de la rehabilitación. Los resultados predominantes evaluados en pacientes con prótesis de cadera incluyeron la puntuación de cadera de Harris (*Harris Hip Score* – HHS) y las actividades de la vida diaria, mientras que la amplitud de movimiento (*Range of Movement – ROM*) y la función de la rodilla se evaluaron en pacientes con prótesis de rodilla.

Entre las intervenciones de enfermería para pacientes con prótesis de cadera, el cinturón elástico de tracción y los cuidados de enfermería de evaluación del dolor realizados por enfermeras y personal médico durante 6 meses (DM: 16,09; intervalo de confianza [IC] del 95%: 8,45–23,73)^(3)^, la enfermería predictiva realizada por enfermeras y equipos de cirugía ortopédica durante 3 meses (DM: 12,1; IC del 95%: 6,35–17,85)^(38)^, y la CNC proporcionada por enfermeras, médicos y terapeutas de rehabilitación durante 3 meses (DM: 8,41; IC del 95%: 4,42–12,40)^(37)^ presentaron las mayores DM en la puntuación de Harries en la cadera. Además, la enfermería predictiva mostró las mayores DM en las actividades de la vida diaria (DM: 13,2; IC del 95%: 6,93–19,47)^(38)^.

Entre las intervenciones de enfermería para pacientes con prótesis de rodilla, los cuidados de enfermería perioperatorios realizados por enfermeras ortopédicas en 1 mes y los cuidados de enfermería para la recuperación mejorada tras la cirugía (*enhanced recovery after surgery* – ERAS) combinados con el entrenamiento de las extremidades realizado por enfermeras (DM: 10,3; IC del 95%: 5,41–15,19)^(27)^ y médicos ortopédicos en 3 meses (DM: 8,2; IC del 95%: 4,30–12,10)^(28)^ mostraron las mayores DM en el movimiento de la rodilla. Además, el ERAS multidisciplinar realizado por un equipo multidisciplinar (enfermeras, cirujanos, anestesistas, nutricionistas, fisioterapeutas y farmacéuticos) en 12 meses (DM: 20; IC del 95%: 10,5–29,5)^(25)^ y el programa de ejercicios en casa basado en una aplicación y dirigido por enfermeras y realizado por enfermeras, desarrolladores de aplicaciones y fisioterapeutas en 3 meses (DM: 14,9; IC del 95%: 7,82–21,98)^(36)^ mostraron las mayores DM en la amplitud de movimiento. Además, el programa de ejercicio en casa basado en una aplicación y dirigido por enfermeras mostró una DM en el dolor (DM: –2,93; IC del 95%: –1,54 a –4,32)^(36)^.

### Resultados del Metanálisis

Teniendo en cuenta la significativa variabilidad de los resultados, el metanálisis se realizó empleando un modelo de efectos aleatorios. Las Figuras 4 y 5 ilustran los resultados del metanálisis realizado sobre la DME de los resultados de rehabilitación tras las intervenciones de enfermería en pacientes con prótesis de cadera y rodilla. Entre los pacientes con prótesis de cadera, los resultados indicaron que las intervenciones de enfermería comparadas con la atención habitual pueden aumentar significativamente la puntuación de Harris en la cadera (DME: 0,734; IC del 95%: 0,521–0,948; *p* < 0,001; ^I2^ = 51,32) y las actividades de la vida diaria (DME: 0,685; IC del 95%: 0,452–0,918; *p* < 0,001; ^I2^ = 56,43). Entre los pacientes con prótesis de rodilla, los resultados mostraron que las intervenciones de enfermería comparadas con la atención habitual pueden aumentar significativamente la amplitud de movimiento (DME: 0,773; IC del 95%: 0,460–1,086; *p* < 0,001; ^I2^ = 60,98) y la función de la rodilla (DME: 0,802; IC del 95%: 0,599–1,006; *p* < 0,001; ^I2^ = 0,001) y disminuir el dolor de rodilla (DME: –0,730; IC del 95%: –0,953 a –0,507; *p* < 0,001; ^I2^ = 53,17).

**Figura 4:** Metaanálisis realizado sobre las diferencias medias estandarizadas de los resultados de la rehabilitación tras intervenciones de enfermería en pacientes con prótesis de cadera.

**Figura 5:** Metaanálisis realizado sobre las diferencias medias estandarizadas de los resultados de la rehabilitación tras intervenciones de enfermería en pacientes con prótesis de rodilla.

La Tabla 3 describe los perfiles GRADE asociados con el efecto de las intervenciones de enfermería en la rehabilitación de pacientes tras una artroplastia de rodilla y cadera. La evaluación reveló que la certeza relacionada con el resultado de la puntuación de Harris era baja, y que la de las actividades de la vida diaria, la función de la rodilla, la amplitud de movimiento y el dolor de rodilla era moderada.

**Tabla 3:** Perfiles GRADE relacionados con el efecto de las intervenciones de enfermería en la rehabilitación de pacientes tras una artroplastia de rodilla y cadera – Huai’an, JS, China, 2024.

Resultados	Riesgo de sesgo	Inconsistencia	Indireccionalidad	Imprecisión	Sesgo de publicación	Número (intervención/control)	DME (95%IC)	Certeza
Puntuación Harris de cadera	No es importante	No es importante	Importante^a^	No es importante	Se sospecha fuertemente la existencia de sesgo de publicación^b^	442/441	0,734 (95% IC: 0,521–0,948)	⨁⨁◯◯ Bajo
Actividades de vida diaria	No es importante	No es importante	No es importante	No es importante	Se sospecha fuertemente la existencia de sesgo de publicación^b^	421/419	0,685 (95% IC: 0,452–0,918)	⨁⨁⨁◯ Moderado
Función de rodilla	No es importante	No es importante	Importante^a^	No es importante	No hay	201/200	0,802 (95% IC: 0,599–1,006)	⨁⨁⨁◯ Moderado
Amplitud de movimiento	No es importante	No es importante	No es importante	No es importante	Se sospecha fuertemente la existencia de sesgo de publicación^b^	279/278	0,773 (95% IC: 0,460–1,086)	⨁⨁⨁◯ Moderado
Dolor de Rodilla	No es importante	No es importante	No es importante	No es importante	Se sospecha fuertemente la existencia de sesgo de publicación^b^	416/415	−0,730 (95% IC: −0,953 to −0,507)	⨁⨁⨁◯ Moderado

^a^Rebajada por su carácter de indireccionalidad en el país.

^b^Se detectó un sesgo de publicación mediante las pruebas de Egger y Begg (*p* < 0,05).

## DISCUSIÓN

La mayoría de los estudios (81%) incluidos en esta revisión se realizaron después de 2020. Esto indica que se trata de un tema interesante, que ha atraído la atención de los investigadores en los últimos años. La mayoría de los estudios (73,7%) utilizaron el ECR como diseño de estudio válido para evaluar esta efectividad. La evaluación de la calidad demostró que la mayoría de los trabajos revisados tienen una calidad adecuada.

En los estudios revisados se utilizaron diversas intervenciones de enfermería para la rehabilitación de los pacientes sometidos a prótesis de rodilla y cadera, que incluían enfermería clínica, intervenciones basadas en la evidencia, programas dirigidos por enfermeras, enfermería a distancia, autocuidados, enfermería de rehabilitación sin dolor, cuidados perioperatorios de enfermería, enfermería especializada, enfermería individualizada, enfermería progresiva, CNC, enfermería predictiva y cuidados de enfermería en combinación con otros métodos. Además, la duración de las intervenciones fue diferente en estos estudios, de 7 días a 12 meses. De los 19 estudios revisados, 17 mostraron que las intervenciones de enfermería pueden mejorar la rehabilitación de los pacientes tras las operaciones de prótesis de rodilla y cadera. Sólo dos estudios sobre programas de enfermería a distancia no mostraron asociaciones significativas entre las intervenciones de enfermería y la rehabilitación en pacientes con reemplazo de rodilla. La eficacia de las diversas intervenciones de enfermería fue diferente.

Entre las intervenciones de enfermería para pacientes con prótesis de cadera, el cinturón elástico de tracción y los cuidados de enfermería de evaluación del dolor realizados por enfermeras y personal médico en 6 meses^(3)^, la enfermería predictiva realizada por enfermeras y equipos quirúrgicos ortopédicos en 3 meses^(38)^ y la CNC realizada por enfermeras, médicos y terapeutas de rehabilitación en 3 meses^(37)^ presentaron las mayores DM en la puntuación de Harris de cadera (*Harris Hip Score -HHS*), respectivamente. Además, la enfermería predictiva mostró las mayores DM en las actividades de la vida diaria^(38)^.

Yu et al. combinaron los cuidados de enfermería con el uso de una banda elástica de tracción para mejorar la rehabilitación de pacientes con prótesis de cadera^(3)^. Un equipo, formado por enfermeras y personal médico, llevó a cabo esta intervención durante 6 meses. lo que afectó significativamente a la HHS de los pacientes. La formación convencional en rehabilitación, que incluye educación sanitaria y orientación médica, favorece la recuperación postoperatoria, pero a menudo provoca resultados subóptimos^(40)^. Los estudios indican que la aplicación de un cinturón de tracción elástico de fabricación propia combinado con cuidados de enfermería estandarizados durante el entrenamiento de rehabilitación afecta significativamente a los resultados de la rehabilitación entre los pacientes tras una artroplastia de cadera^(3)^.

Guo et al. utilizaron intervenciones de enfermería predictivas para la rehabilitación de pacientes ancianos. Enfermeras y equipos de cirugía ortopédica llevaron a cabo esta intervención^(38)^. Mejoró significativamente la HHS y las actividades de la vida diaria de los pacientes^(38)^. La estrategia de enfermería predictiva emplea una metodología basada en la evidencia para predecir las necesidades de los pacientes y prevenir complicaciones mediante la identificación precoz de posibles riesgos durante el proceso asistencial^(41)^. Este enfoque abarca evaluaciones integrales del paciente, como los aspectos físicos, psicológicos y sociales, y utiliza análisis y herramientas predictivas para categorizar a los pacientes según sus niveles de riesgo^(42)^. Dicha categorización permite aplicar intervenciones individualizadas, monitorizar de forma continua y detectar precozmente el deterioro clínico, mejorando así los resultados de los pacientes^(43)^. En los últimos años, ha aumentado la importancia de los enfoques de atención individualizada, especialmente en el contexto de procedimientos médicos complejos^(44,45)^.

Guo et al. también aplicaron un modelo de CNC a pacientes ancianos sometidos a artroplastia total de cadera^(37)^. Enfermeras, médicos y terapeutas de rehabilitación proporcionaron esta intervención durante 3 meses. Aumentó significativamente la HHS de los pacientes. En el CNC, las necesidades sanitarias del paciente y los recursos disponibles se evalúan antes del alta hospitalaria. Este enfoque incluye educación sanitaria, instrucciones al alta, seguimientos telefónicos y visitas domiciliarias realizadas por un equipo de enfermeras de prácticas experimentadas y diversos profesionales sanitarios^(37)^. Mientras tanto, los cuidados de enfermería tradicionales se centran principalmente sólo en la fase perioperatoria, lo que expone a los pacientes a varios riesgos potenciales tras el alta^(37)^.

Entre las intervenciones de enfermería para pacientes sometidos a prótesis de rodilla, los cuidados de enfermería perioperatorios realizados por enfermeras ortopédicas durante un mes^(27)^ y los cuidados de enfermería para una mayor recuperación tras la cirugía (*Enhanced recovery after surgery - ERAS*) combinados con el entrenamiento de las extremidades realizado por enfermeras y médicos ortopédicos en 3 meses^(28)^ mostraron las DM más altas para el movimiento de la rodilla. Además, la ERAS realizado por un equipo multidisciplinar, formado por enfermeras, cirujanos, anestesistas, nutricionistas, fisioterapeutas y farmacéuticos, en 12 meses^(25)^ y un programa de ejercicios en casa basado en una aplicación y dirigido por enfermeras, desarrolladores de aplicaciones y fisioterapeutas en 3 meses^(36)^ mostraron las mayores DM en la amplitud de movimiento. Además, los programas de ejercicio en casa basados en aplicaciones y dirigidos por enfermeras mostraron las mayores DM en dolor^(36)^.

Sun et al.^(27)^ investigaron el efecto de la enfermería perioperatoria en la rehabilitación funcional de la rodilla. Esta intervención fue realizada por enfermeras ortopédicas durante 1 mes^(27)^. La eficacia de esta intervención puede deberse a la participación de enfermeras especializadas^(27)^. La enfermería perioperatoria destaca el papel crucial de las enfermeras especializadas en el proceso de tratamiento, lo que refuerza su sentido de la responsabilidad e identidad dentro de la profesión de enfermería y contribuye a la eficacia de los equipos médicos en la mejora de los resultados del tratamiento^(46)^. Esta intervención proporciona una base en la medicina basada en la evidencia que respalda el avance de las prácticas de enfermería^(27)^.

Li et al. también combinaron la enfermería ERAS con el entrenamiento de las extremidades para aumentar la rehabilitación de la función de la rodilla. Un equipo de enfermeras y médicos ortopédicos llevó a cabo esta intervención durante 3 meses^(28)^. En este estudio, el ejercicio de las extremidades se añadió al programa ERAS como una intervención basada en la evidencia, utilizando un equipo especializado para mejorar la rehabilitación. El ERAS integra prácticas de enfermería tradicionales en todas las fases perioperatorias y optimiza estos métodos para lograr los resultados deseados^(47)^. Garantiza una recuperación postoperatoria continua mediante la creación de un equipo de enfermería especializado. Este equipo recibe una formación sistemática en el tratamiento de la enfermedad y en técnicas de cuidados postoperatorios, lo que les permite proporcionar cuidados programados y estandarizados a los pacientes antes y después de la operación^(28)^. Además, el ejercicio precoz de las extremidades reduce el riesgo de trombosis venosa, favorece la reparación de los tejidos adyacentes y disminuye el dolor relacionado con las lesiones articulares^(28)^. La mejora del dolor postoperatorio puede estar asociada al hecho de que los ejercicios postoperatorios de las extremidades mejoran el ROM de la articulación de la rodilla, aumentan la fuerza muscular y mejoran la resistencia y la coordinación articulares^(47)^. Los resultados revelan que la incorporación de ejercicios de las extremidades en el marco del ERAS también puede mejorar la funcionalidad de la rodilla^(47)^.

Liu et al.^(25)^ investigaron el efecto de ERAS con colaboración multidisciplinar en los resultados de enfermería^(25)^. Un equipo multidisciplinar, formado por cirujanos, anestesistas, enfermeras, fisioterapeutas, nutricionistas y farmacéuticos, llevó a cabo esta intervención durante 12 meses. Esta intervención aumentó significativamente el ROM en los pacientes^(25)^. La necesidad de colaboración multidisciplinar en los protocolos de ERAS es crucial, ya que implica a varios profesionales sanitarios durante la fase perioperatoria^(48)^. Esta estrategia garantiza que la asistencia esté estandarizada y adaptada, se base en las mejores pruebas actuales y se ajuste a las necesidades específicas del paciente^(49)^. Además, la colaboración interdisciplinaria mejora la interacción y el aprendizaje entre los profesionales sanitarios, los pacientes y sus familias, lo que se traduce en una atención coherente y de alta calidad y aumenta la satisfacción y el cumplimiento por parte del paciente^(50)^.

Bak and Uhm^(36)^ utilizaron un programa de ejercicios en casa basado en una aplicación y dirigido por enfermeras para la rehabilitación de pacientes sometidos a prótesis de rodilla y observaron que esta intervención tenía un efecto significativo en el ROM y el dolor de rodilla de los pacientes^(36)^. Enfermeras, desarrolladores de aplicaciones y fisioterapeutas llevaron a cabo esta intervención durante 3 meses. El ejercicio postoperatorio es una parte integral del proceso de rehabilitación destinado a restaurar la función articular. Las investigaciones indican que los ejercicios tempranos en la primera semana del postoperatorio producen resultados favorables^(36)^. La atención sanitaria móvil, que supera las limitaciones geográficas y temporales, se utiliza para compartir información sanitaria y facilitar la interacción de los pacientes con los profesionales sanitarios^(51,52)^. Este método se empleó con éxito para la rehabilitación de pacientes tras una prótesis de rodilla. Sin embargo, cabe destacar que esta formación debe basarse en un programa dirigido por enfermeras para obtener resultados adecuados. En este enfoque, las enfermeras desempeñan un papel fundamental al proporcionar cuidados integrales, defensa y liderazgo. Las enfermeras tienen capacidades y perspectivas distintas que les permiten atender a las personas en su totalidad y formar una asociación esencial entre los cuidados compasivos y las prácticas basadas en la evidencia^(53)^. Las medidas dispersas no pueden asociarse a éxitos significativos. Así, dos estudios sobre enfermería a distancia que utilizaron la autogestión del dolor dirigida por el paciente y la navegación de los cuidados postoperatorios basada en la IM no demostraron resultados significativos^(10,39)^.

En conjunto, los resultados de estos estudios indican la importancia de los servicios de enfermería especializados en combinación con otros servicios de rehabilitación entre los pacientes sometidos a prótesis de rodilla y cadera, lo que puede deberse a que las prótesis de rodilla y cadera requieren una atención especial. Por lo tanto, incluso después del alta, los ejercicios y las intervenciones deben continuar bajo la supervisión de un equipo especializado hasta que el paciente recupere la capacidad funcional adecuada.

Según la evaluación, la certeza asociada con el resultado de la HHS fue baja, y la certeza relacionada con los resultados de las actividades de la vida diaria, la función de la rodilla, el ROM y el dolor de rodilla fue moderada. Es muy probable que la investigación adicional exhiba un efecto importante sobre nuestra confianza en la estimación del efecto y es probable que cambie la estimación para los resultados con certeza baja.

Como limitación, el diseño de los programas de intervención difirió entre los diferentes estudios, lo que también puede introducir algunos sesgos. Además, el tiempo de seguimiento fue diferente entre los estudios. Además, en los estudios revisados no se investigaron las diferencias entre los distintos géneros y edades. Como otra limitación, no se incluyeron en la revisión los trabajos en lengua no inglesa.

## CONCLUSIÓN

La mayoría de los estudios revisados revelaron que las intervenciones de enfermería pueden mejorar la rehabilitación de los pacientes después del reemplazo de rodilla y cadera. La efectividad de las diversas intervenciones de enfermería varió. Las intervenciones de enfermería con mayor efectividad incluyeron la enfermería predictiva, la enfermería continua, la enfermería de evaluación del dolor combinada con un cinturón elástico de tracción, la enfermería perioperatoria, la enfermería ERAS en combinación con el entrenamiento de las extremidades y un programa de ejercicios domiciliarios basado en una aplicación dirigida por enfermeras. En resumen, las pruebas indican que la atención individualizada y las estrategias basadas en la evidencia en combinación con diversos ejercicios y entrenamiento (por ejemplo, ejercicios de rehabilitación, ejercicios postoperatorios de las extremidades, ejercicios de las extremidades en el marco del ERAS, ejercicios tempranos en la primera semana postoperatoria), que son proporcionados por un equipo multidisciplinar con el liderazgo de una enfermera, pueden afectar significativamente a la rehabilitación física entre los pacientes sometidos a reemplazo de rodilla y cadera. Además, estas intervenciones pueden tener más éxito cuando se realizan como planes continuos desde el preoperatorio hasta el postoperatorio y después del alta. Se pueden emplear aplicaciones móviles, teléfonos, visitas presenciales y otros métodos de comunicación para continuar con estas medidas. Por lo tanto, en los próximos estudios se desarrolla un modelo novedoso de programas de enfermería basado en medidas integrales y continuas con un enfoque interactivo combinado con tecnología remota. En los próximos estudios se evaluará la eficacia de las nuevas intervenciones de enfermería en comparación con las intervenciones de enfermería anteriores. Además, la eficacia de estas intervenciones se compara entre varios grupos, en términos de edad, sexo y otras características demográficas.

## Data Availability

Los datos utilizados para obtener los hallazgos de este estudio están disponibles a pedido razonable del autor correspondiente.

## MATERIAL SUPLEMENTARIO

El siguiente material suplementario está disponible en línea:

Table S1 – The search strategy in the present study – Huai’an, JS, China, 2024.
